# Evaluation of a PSA and transrectal prostate ultrasound video-based machine learning model as a tool for prostate cancer diagnosis

**DOI:** 10.3389/fonc.2025.1590396

**Published:** 2025-09-08

**Authors:** Yanhong Du, Anli Zhao, Maoliang Zhang, Zhengping Wang, Liyan Hu, Xiaoyang Qi

**Affiliations:** Department of Ultrasound, The Affiliated Dongyang Hospital of Wenzhou Medical University, Dongyang, Zhejiang, China

**Keywords:** prostate cancer, PSA, machine learning, prostate ultrasound video, SHAP

## Abstract

**Objective:**

To develop a machine learning-based model incorporating prostate-specific antigen (PSA) levels and prostate ultrasound video clips for diagnosing prostate cancer.

**Methods:**

The study enrolled 928 participants, of whom 429 had prostate cancer and 499 other non-prostate cancers. Univariate and multivariate analyses of serological indices were conducted to detect significant variables. From this cohort, 742 patients were randomly chosen for model validation, while the other 186 were employed to evaluate the accuracy and reliability of the model. Seven features were extracted from ultrasound video clips and combined with PSA and other clinical indicators. Predictive models were established using six machine learning algorithms and receiver operating characteristic (ROC) curves were used to determine the optimal model. SHapley Additive exPlanations (SHAP) was utilized to visualize feature importance in the best-performing model.

**Results:**

All six of the evaluated machine learning models performed favorably, with area under the ROC curve (AUC) values in the test set ranging from 0.800 to 0.881. Of these models, the XGBoost model achieved the most promising performance, significantly surpassing that of the other models (P < 0.05). SHAP visualization revealed that PSA, prostatic volume(PV), age, wavelet.LHL.firstorder. Median, wavelet.HLH.glszm.ZoneEntropy, and original.shape.MinorAxisLength were the most influential features in the XGBoost model.

**Conclusion:**

The developed machine learning models demonstrated significant potential for prostate cancer diagnosis. Among them, the XGBoost model outperformed the others, highlighting its superior predictive capability.

## 1 Introduction

Prostate cancer(PCa) ranks second in global male malignancies, after only lung cancer (1). In China, both diagnoses and deaths associated with prostate cancer have been increasing steadily in recent years (2). Per the World Health Organization, in 2020, China reported an incidence rate of 15.6 per 100,000 individuals, with more than 110,000 new diagnoses and more than 50,000 deaths, making it a significant public health concern (3). The clinical state of prostate cancer in China differs considerably from that in Western countries. Multi-center studies indicate that only one in three cases are localized at diagnosis, with most being at intermediate or advanced stages, resulting in poorer prognosis compared to Western countries (4). Studies have shown that over half of patients exhibit bone metastases at initial diagnosis, often accompanied by complications such as bone pain, movement disorders, pathological fractures, and spinal cord compression (5).

Early screening, early diagnosis, and early treatment are all invaluable strategies for improving survival rates among prostate cancer patients. Elevated PSA levels are commonly related to prostate cancer progression. Currently, PSA testing is the primary method for prostate cancer screening, with a diagnostic threshold of 4.0 ng/mL (6). Patients with PSA levels exceeding this threshold generally receive transrectal ultrasound (TRUS)-guided prostate biopsy procedures. However, this invasive procedure can cause psychological distress in some patients. Consequently, non-invasive diagnostic methods are increasingly being explored.

Recently, radiomics and machine learning have gained prominence in medical imaging applications. Radiomics involves extracting a large number of quantitative features from CT, MRI, or PET imaging, capturing tumor heterogeneity beyond traditional morphological assessment and associated subjective limitations pertaining to the visual assessment of target lesions (7, 8). Traditional imaging primarily evaluates tumor size, location, and shape, whereas radiomics provides deeper insights into tumor texture and internal structure. Machine learning, as an interdisciplinary field encompassing probability theory, statistics, and algorithmic complexity, convex analysis, approximation theory, and other areas, enables computers to simulate human learning behaviors, refine knowledge structures, and enhance predictive accuracy. Radiomics offers an ideal source of abundant data to fuel machine learning applications as it provides many imaging features for analysis. In the context of radiomics, machine learning is used to analyze and model extracted imaging features. The synergy between radiomics and machine learning facilitates precise medical image analysis, improving diagnostic accuracy, treatment decision-making, and non-invasive tumor assessment. These strategies have been extensively applied when diagnosing prostate cancer, invasiveness assessment, and clinical decision-making (7).

This study aimed to integrate serum PSA-related indicators with TRUS imaging features, employing six machine learning algorithms to develop predictive models for early, non-invasive prostate cancer diagnosis suitable for facilitating timely intervention and patient treatment.

## 2 Materials and methods

### 2.1 Participants

**Figure 1:**
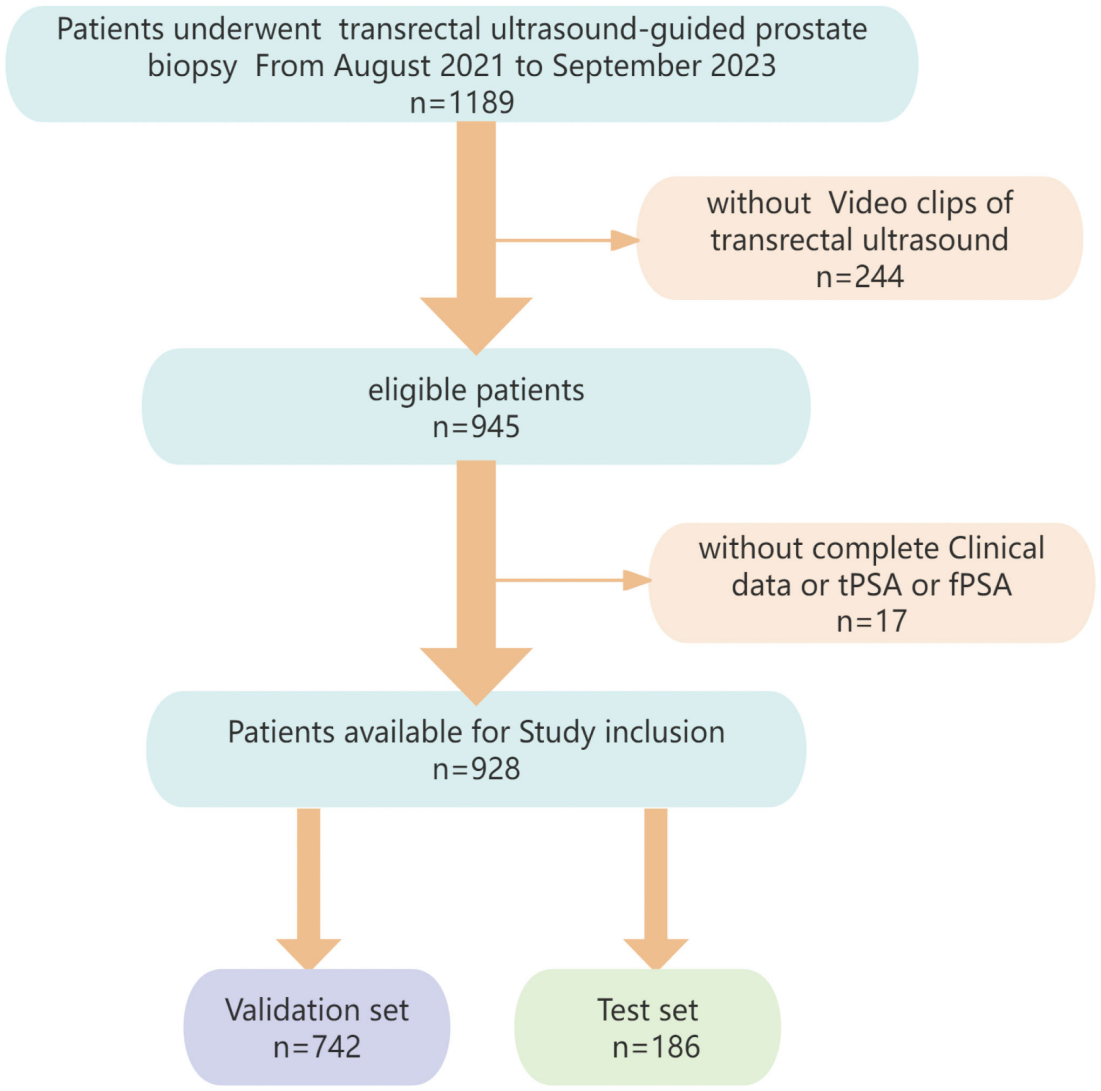
Flowchart showing the process of patient selection.

The data of 928 cases (429 PCa, 499 non-PCa) from Dongyang Hospital of Wenzhou Medical University were retrospectively analyzed (August 2021–September 2023). Inclusion criteria: (1)preoperative analyses of PSA (total PSA [tPSA], free PSA [fPSA], f/tPSA ratio); (2) TRUS examination; (3) histopathologically confirmed disease by transperineal biopsy. Exclusion criteria: (1) prior prostate therapies (e.g., hormonal/radiation therapy); (2) incomplete clinical/imaging data. Participants were randomized into training/validation (n=742) and test (n=186) cohorts. The institutional ethics committee provided approval for the study protocol. **Figure 1** illustrates the participant selection process.

### 2.2 Data acquisition

1) Ultrasound equipment and acquisition parameters:

Instrumentation: The Esaote MyLab™ ClassC color Doppler ultrasound diagnostic instrument equipped with a TRT 33 transrectal biplane ultrasound probe (frequency range: 3 – 13 MHz) was used for TRUS examinations.

Scan mode setting: B-mode grayscale imaging was used with adjustment of the depth to cover the prostate and surrounding tissues (default depth, 59 mm, default gain intensity, 60%).

Patient position: The patient was placed in the lithotomy position. After insertion of the probe, fine-tuning was performed to ensure that the symmetry axis between the urethra and the gland was clearly visible in the image.

Standard measurement of prostate size in two-dimensional ultrasound: The long diameter section of the prostate (through the long axis of the urethra, showing both the internal opening of the urethra and the tip of the prostate) was assessed by measuring the upper and lower diameters (L) and anteroposterior diameters (H), with the anteroposterior diameter representing the maximum diameter perpendicular to the long diameter in the middle and upper one-third of the prostate area. For determination of the maximum cross-sectional area of the prostate (both left and right lobes can be displayed), the left and right diameters (W) were measured, and the prostate volume (PV) was calculated as: PV = 0:52*L*W*H (9).

The standard used for obtaining ultrasound video was determined by an ultrasound physician with over 10 years of experience. The scan video of the prostate cross-section was conducted using constant speed for 5 – 10 seconds, with scanning from the internal opening of the bladder and urethra to the tip of the prostate and including 2 – 5 mm of normal tissues on both sides, using the DCM format.

Probe motion speed: Constant linear sweep at 0.5 - 1.6 cm/sec.

Frame rate: 30 fps (DCM format, 150–300 frames/clip).

Frame selection: Consecutive frames without exclusion, as radiomic features were extracted from the entire video volume. Motion artifacts were minimized by standardized operator training and real-time quality checks.

Representative frames: Not applicable, as features were derived from 3D volumetrics (not 2D frames).

2) PSA analyses: Venous blood samples were collected before TRUS, cystoscopy, or any other procedures that could influence serum PSA levels. The tPSA and fPSA concentrations were measured using the Roche E602 and E801 automatic chemiluminescence immunoanalyzers (Roche, Germany). The PSA density (PSAD) was calculated as follows: tPSA/PV.

3) Prostate biopsy: Systematic 12-core biopsies were conducted with TRUS guidance and an 18G Magnum biopsy gun.

### 2.3 Manual segmentation and feature extraction

Manual annotation was performed using 3D Slicer (v 5.0.3). Three experienced radiologists with >5 years’ experience in TRUS diagnosis independently delineated the complete prostate as the region of interest (ROI). To maintain objectivity, patient identifiers were removed, each case was assigned a numerical code, and data were randomized to ensure blinding. A 3D Slicer with the SlicerRadiomics extension was used for extraction of radiomic features (10). Features extracted for this study consisted of shape-based,first-order statistical, gray-level run length matrix (GLRLM), gray-level size zone matrix (GLSZM), gray-level dependence matrix (GLDM), gray-level co-occurrence matrix (GLCM), neighborhood gray-tone difference matrix (NGTDM), and wavelet-transformed features. Features with intra- and inter-class correlation coefficients (ICCs) ≥ 0.75 were retained for further analysis.

### 2.4 Feature screening

Feature selection was performed using R (v 4.2.1) and was divided into two parts: PSA-related indicator selection and radiomic feature selection.

PSA-related indicator selection: Univariate and multivariate logistic regression approaches were utilized to evaluate age, prostate volume (PV), tPSA, fPSA, PSAD, f/tPSA, and (f/tPSA)/PCAD. Significant variables (p < 0.05) were included in the final model.

Radiomics feature screening: Features were normalized using z-scores for standardization of data distributions. Three stages were involved in feature selection in the training cohort: (1) ICC Filtering: Features with ICC values below 0.75 were eliminated to reduce redundancy; (2) Statistical Filtering: Features with a p-value > 0.05 in the Mann-Whitney U test were excluded, ensuring retention of only statistically relevant features; (3) LASSO (Least Absolute Shrinkage and Selection Operator) Regression: The LASSO method was implemented with the R glmnet. AN optimal penalty coefficient (λ) was established through 10-fold cross-validation, and features with nonzero coefficients were retained. The ‘one standard error” rule (lambda.1se) was adopted to enhance the robustness of the model; this selects the largest λ value within the range of one standard error from the minimum cross-validation error, which prioritizes sparse but stable feature sets (11, 12).

### 2.5 Machine learning

Using R (v 4.2.1), the dataset was randomly stratified at an 80:20 ratio for validation and testing. Six machine learning algorithms were implemented, including Light Gradient Boosting Machine (LightGBM), Logistic Regression (LR), Decision Tree (DT), Support Vector Machine (SVM), Random Forest (RF), and Extreme Gradient Boosting (XGBoost). Model performance was assessed using accuracy (ACC), sensitivity (SEN), specificity (SPE), positive/negative predictive value (PPV and NPV), and F1-scores. Diagnostic efficacy was explored with receiver operating characteristic (ROC) curves, and the area under the curve (AUC) was determined to identify the best-performing model. The SHapley Additive exPlanations (SHAP) method (13) was used to interpret the optimal model, and feature importance was visualized with the shapviz package in R.

### 2.6 Statistical analysis

R (v 4.2.1) was used to analyze all data. Results that were normally distributed were presented as means ± standard deviation ($\overset{—}{x}$ ± s) and analyzed through independent sample t-tests. Non-normally distributed data are given as median (quartiles) [M (P25, P75)] and were analyzed using the Mann-Whitney U test. Statistical significance was defined by a p < 0.05.

## 3 Results

### 3.1 Baseline characteristics

The study enrolled 928 participants, with 429 diagnosed with prostate cancer and 499 classified in the non-PCa group. Patients were randomly divided into validation and test sets (80:20 ratio), with no significant differences between the groups (**Table 1**).

**Table 1:** Clinicopathological characteristics in the validation and test groups.

Variables	Total (n = 928)	Test (n = 186)	Train (n = 742)	p
Group, n (%)				0.284
Non-PCa	499 (54)	93 (50)	406 (55)	
Pca	429 (46)	93 (50)	336 (45)	
Median (interquartile range)				
Age	72 (67, 77)	71 (67, 76.75)	72 (67, 77)	0.922
PV	42.21 (31.45, 58.57)	44.58 (31.75, 59.38)	41.51 (31.34, 58.09)	0.412
tPSA	8.41 (5.52, 14.25)	8.69 (6.32, 14.89)	8.29 (5.43, 14.05)	0.104
fPSA	1.21 (0.77, 2.07)	1.28 (0.9, 2.17)	1.18 (0.76, 2.03)	0.132
f/tPSA	0.14 (0.1, 0.18)	0.13 (0.1, 0.18)	0.14 (0.1, 0.18)	0.66
PSAD	0.19 (0.13, 0.35)	0.21 (0.13, 0.36)	0.18 (0.12, 0.35)	0.253
(f/tPSA)/PSAD	0.74 (0.31, 1.35)	0.66 (0.29, 1.18)	0.76 (0.32, 1.36)	0.312

PCa, prostate cancer; Non-PCa, Non-prostate cancer; PV, prostate volume; tPSA, total prostate specific antigen; fPSA, free prostate specific antigen; f/tPSA, free prostate specific antigen ratio; PSAD, prostate specific antigen density; (f/tPSA)/PSAD, free prostate specific antigen ratio/prostate specific antigen density.

### 3.2 Feature screening

**Figure 2:**
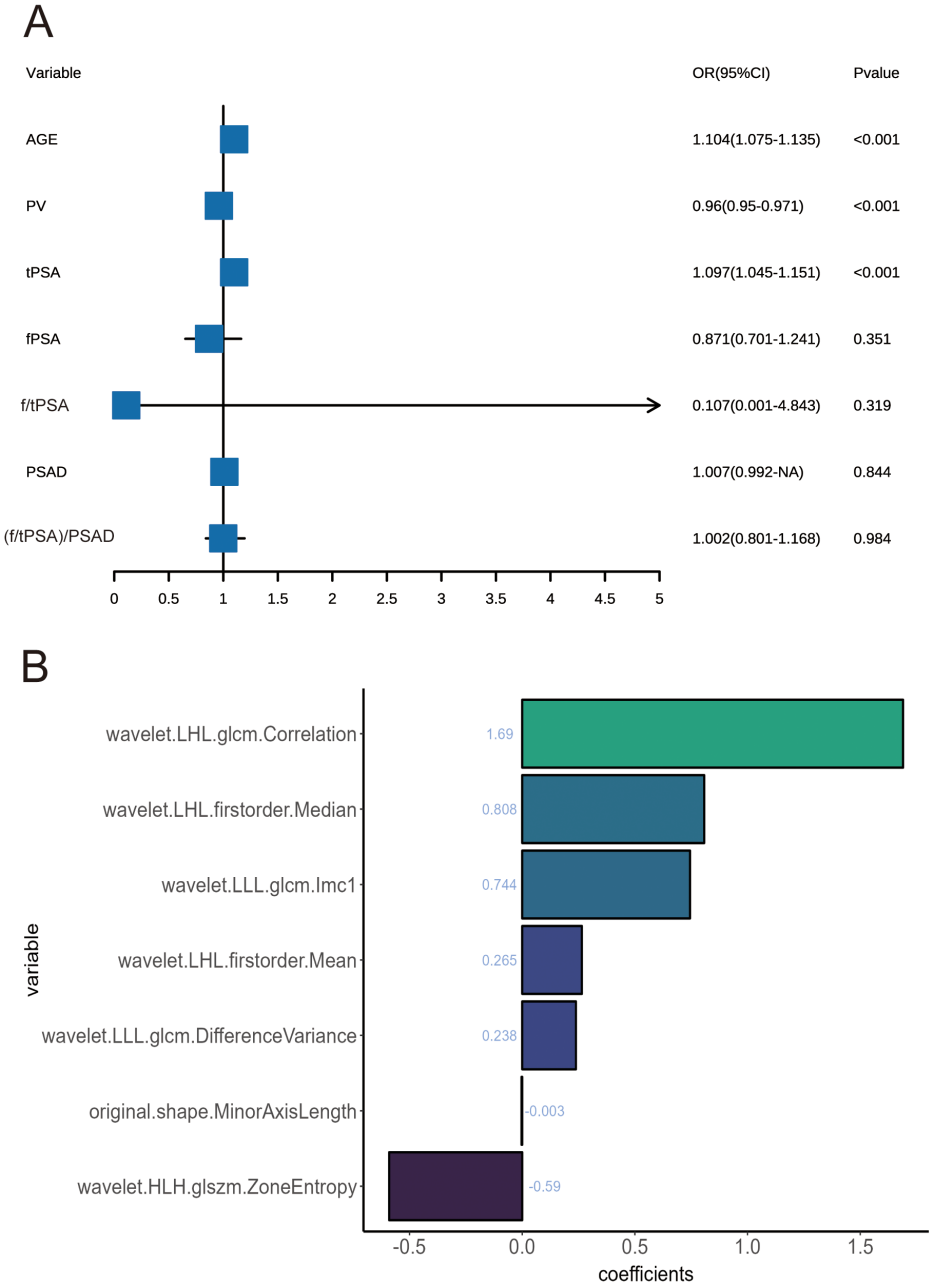
**(A)** Forest plots of influencing factors associated with prostate cancer, as identified in multivariate analyses. **(B)** Feature classification weights.

Univariate and multivariate regression analyses were performed on seven factors when screening for PSA-associated indicators, including age, PV, tPSA, and fPSA (**Table 2**). Multivariate analysis identified age, PV, and PSA as significant predictors (**Figure 2A**).

**Table 2:** Univariate and multivariate analyses.

Characteristics	Uni-B	Uni-SE	Uning-OR	Uni-CI	Uni-Z	Uni-P	Multi-B	Multi-SE	Multi-OR	Multi-CI	Multi-Z	Multi-P
(f/tPSA)/PSAD	-1.012	0.12043	0.363	0.363(0.312 - 0.457)	-8.403	<0.001	0.002	0.09007	1.002	1.001 (0.801 - 1.168)	0.021	0.984
PSAD	3.733	0.46562	41.808	41.80(17.63 - 109.5)	8.017	<0.001	0.007	0.03575	1.007	1.007 (0.992-NA)	0.196	0.844
f/tPSA	-7.076	1.22499	0.001	0.001(0 - 0.009)	-5.777	<0.001	-2.238	2.24513	0.107	0.106 (0.000 - 4.843)	-0.997	0.319
fPSA	0.238	0.04691	1.269	1.269(1.166 - 1.401)	5.078	<0.001	-0.138	0.14804	0.871	0.871 (0.701 - 1.240)	-0.932	0.351
tPSA	0.067	0.00971	1.07	1.07(1.051 - 1.091)	6.93	<0.001	0.093	0.02414	1.097	1.096 (1.044 - 1.150)	3.833	<0.001
PV	-0.017	0.00346	0.983	0.983(0.976 - 0.989)	-4.987	<0.001	-0.041	0.00551	0.96	0.960 (0.949 - 0.970)	-7.378	<0.001
Age	0.09	0.01116	1.095	1.095(1.071 - 1.119)	8.093	<0.001	0.099	0.01364	1.104	1.103 (1.075 - 1.134)	7.25	<0.001

PV, prostate volume; tPSA, total prostate specific antigen; fPSA, free prostate specific antigen; f/tPSA, free prostate specific antigen ratio; PSAD, prostate specific antigen density; (f/tPSA)/PSAD, free prostate specific antigen ratio/prostate specific antigen density.

**Figure 3:**
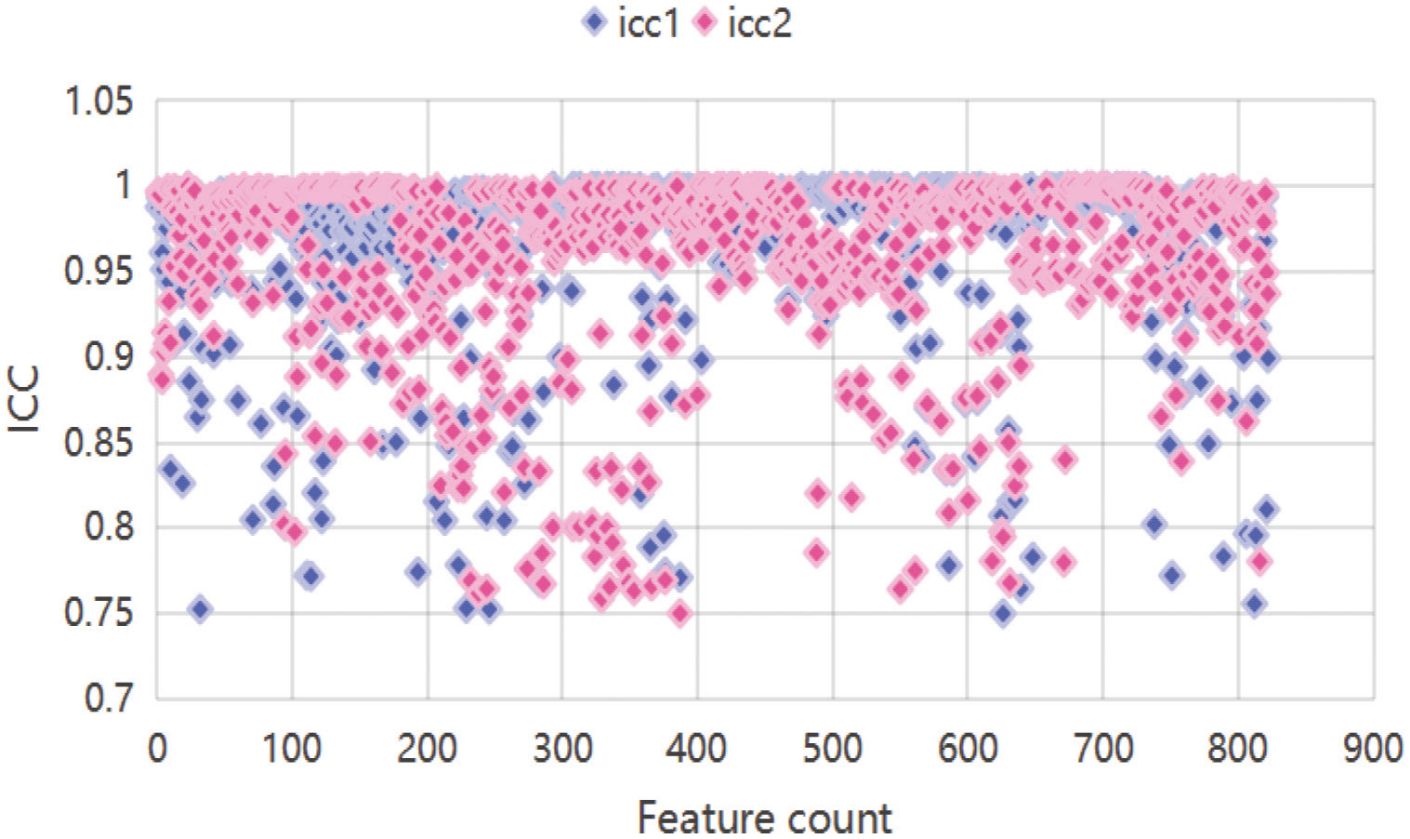
Scatter plot features. icc1 represents inter-reader ICCs; icc2 represents intra-reader ICCs.

**Figure 4:**
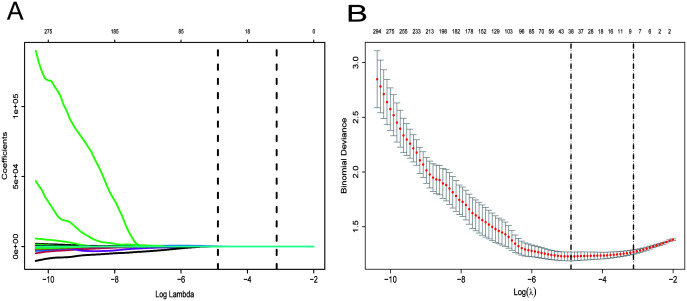
Selection of the optimal penalization coefficient (λ). **(A)** Ten-fold cross-validation for the tuning of feature selection in the LASSO model. **(B)** Ten-fold cross-validation for the tuning of parameter selection in the LASSO model. **(B)** LASSO coefficient solution path for the seven identified features.

When screening for radiomics features, a total of 851 radiomic features were extracted per patient, categorized as follows: 14 shape features, 18 first-order statistical features, 14 GLDM features, 16 GLRLM features, 16 GLSZM features, 24 GLCM features, 5 NGTDM features and ell as 744 wavelet features. After ICC analysis, 822 features with ICC > 0.75 were retained. The ICC values are shown in **Supplementary Table S1** and the scatter plot in **Figure 3**. Subsequent statistical filtering eliminated 392 features, leaving 430 for further refinement. Following LASSO regression, seven features—one shape feature and six wavelet-transformed features—were selected for the final model (**Table 3**). **Figure 2A** presents the selected significant PSA-related indicators, **Figure 2B** shows the feature importance in the validation set, and **Figures 4A, B** illustrates the determination of the optimal penalty coefficient λ.

**Table 3:** Selected LASSO regression-derived radiomics features.

Feature	Image type	Feature class	Feature name	Lambda.1se.coef
1	original	shape	MinorAxisLength	-0.003250686
2	wavelet.LHL	firstorder	Mean	0.264773125
3	wavelet.LHL	firstorder	Median	0.807724427
4	wavelet.LHL	glcm	Correlation	1.690144747
5	wavelet.HLH	glszm	ZoneEntropy	-0.590446784
6	wavelet.LLL	glcm	DifferenceVariance	0.238408355
7	wavelet.LLL	glcm	Imc1	0.744481738

**Figure 5:**
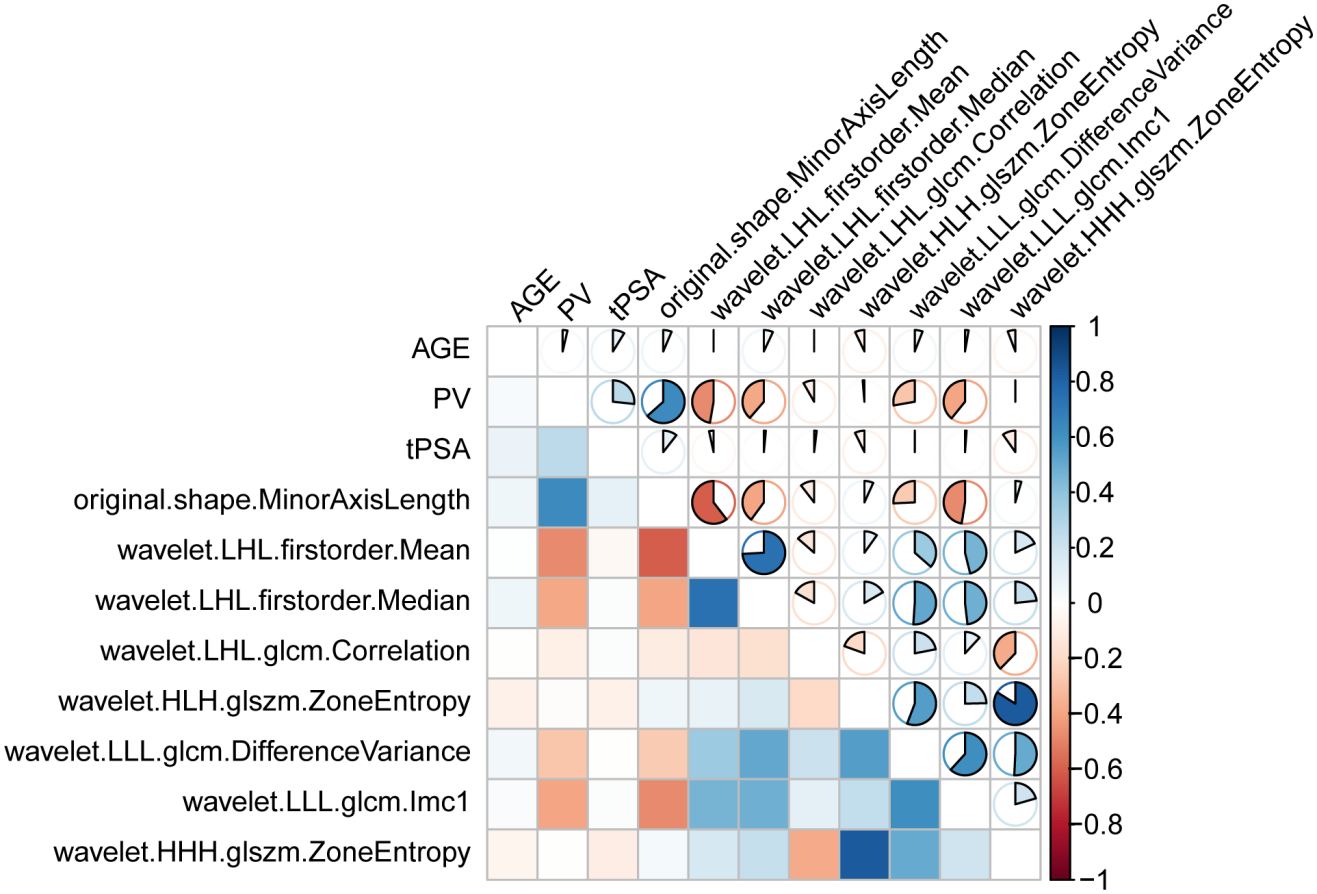
Spearman’s correlation coefficients for the indicated features.

The selected seven key features, combined with age, PV, and tPSA, formed the final model (**Figure 5**). The heatmap in **Figure 5** displays the correlation coefficients of selected features.

### 3.3 Evaluation of model performance

**Figure 6:**
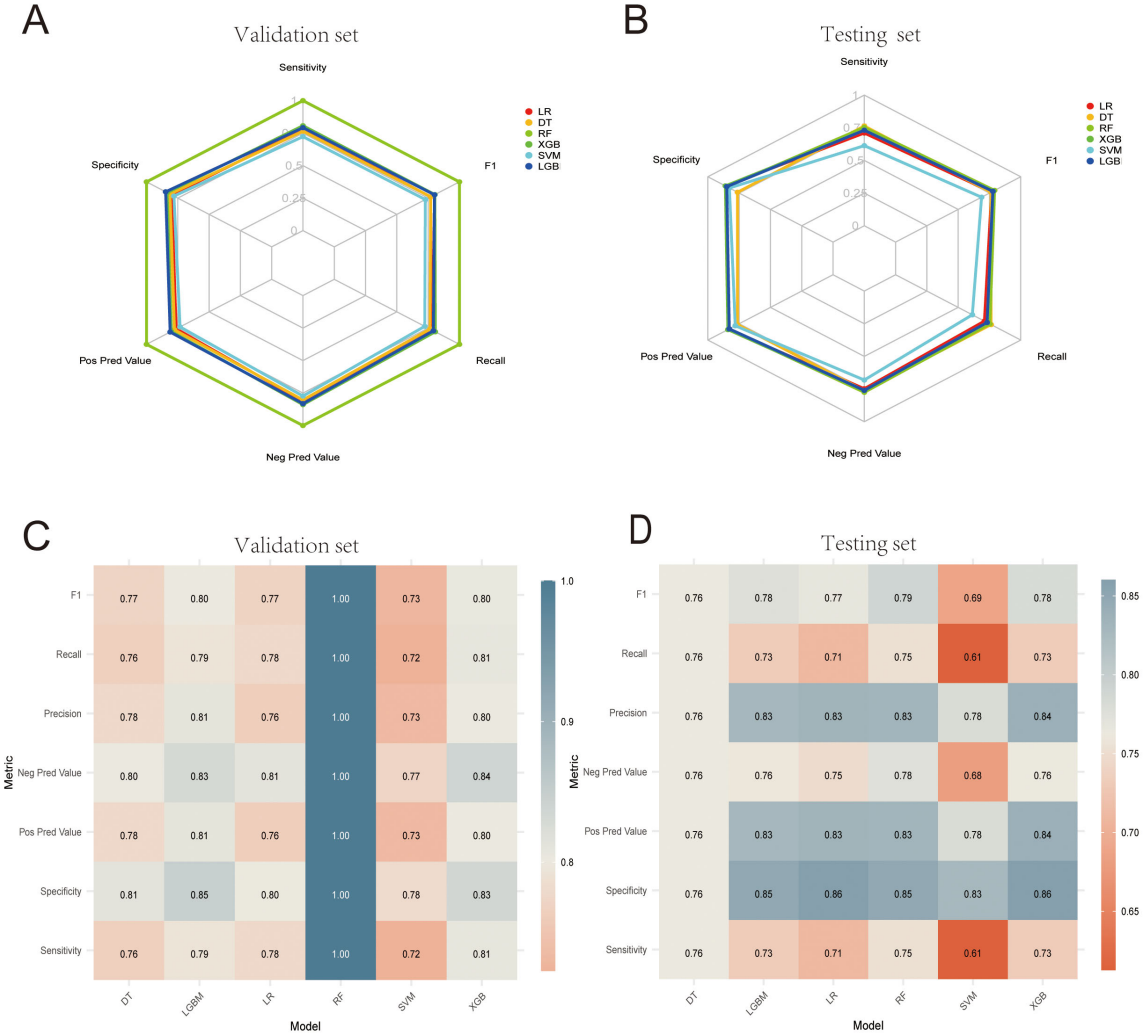
**(A, B)** Machine learning confusion matrices in the internal validation and test cohorts. **(C, D)** Heatmaps corresponding to the machine learning confusion matrices in the internal validation set and test cohorts.

**Figure 7:**
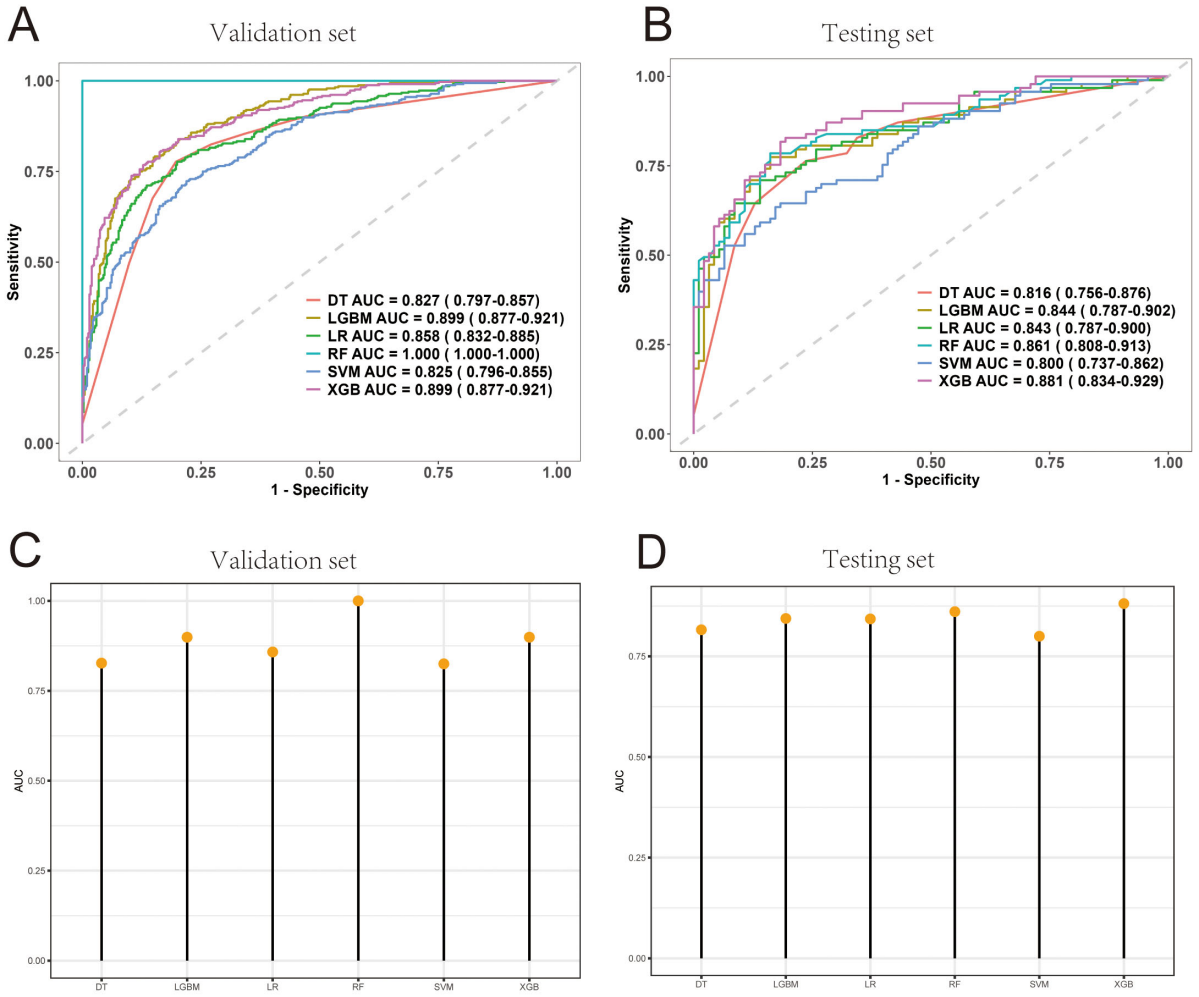
ROC curves for the six evaluated models. **(A, B)** ROC curves for the six models in the internal validation and test cohorts. **(C, D)** AUC value comparisons for the six models in the internal validation and test cohorts.

**Table 4** presents the performance metrics of the six machine learning models, all of which demonstrate strong predictive capabilities, with the AUC ranging from 0.800 to 0.881. The confusion matrices for both the training and test sets are visualized in **Figure 6**. Among these models, XGBoost achieved the highest AUC and exhibited the best overall performance, markedly superior to the other models (P < 0.05). **Figure 7** illustrates the ROC curves and AUCs for the models in the training and test sets.

**Table 4:** Model-specific testing and validation.

Model	Data set	AUC	Accuracy	Sensitivity	Specificity	Pos Pred Value	Neg Pred Value	F1
LR	Validation set	0.858	0.789	0.777	0.800	0.763	0.812	0.770
	Testing set	0.843	0.785	0.710	0.860	0.835	0.748	0.767
DT	Validation set	0.827	0.789	0.765	0.813	0.777	0.803	0.771
	Testing set	0.816	0.763	0.763	0.763	0.763	0.763	0.763
RF	Validation set	1.000	1.000	1.000	1.000	1.000	1.000	1.000
	Testing set	0.861	0.801	0.753	0.849	0.833	0.775	0.791
XGB	Validation set	0.899	0.821	0.807	0.835	0.802	0.839	0.804
	Testing set	0.881	0.796	0.731	0.860	0.840	0.762	0.782
SVM	Validation set	0.825	0.752	0.723	0.781	0.732	0.773	0.728
	Testing set	0.800	0.720	0.613	0.828	0.781	0.681	0.687
LGBM	Validation set	0.825	0.819	0.792	0.847	0.811	0.831	0.801
	Testing set	0.844	0.790	0.731	0.849	0.829	0.760	0.777

### 3.4 Optimal model SHAP visualization

**Figure 8:**
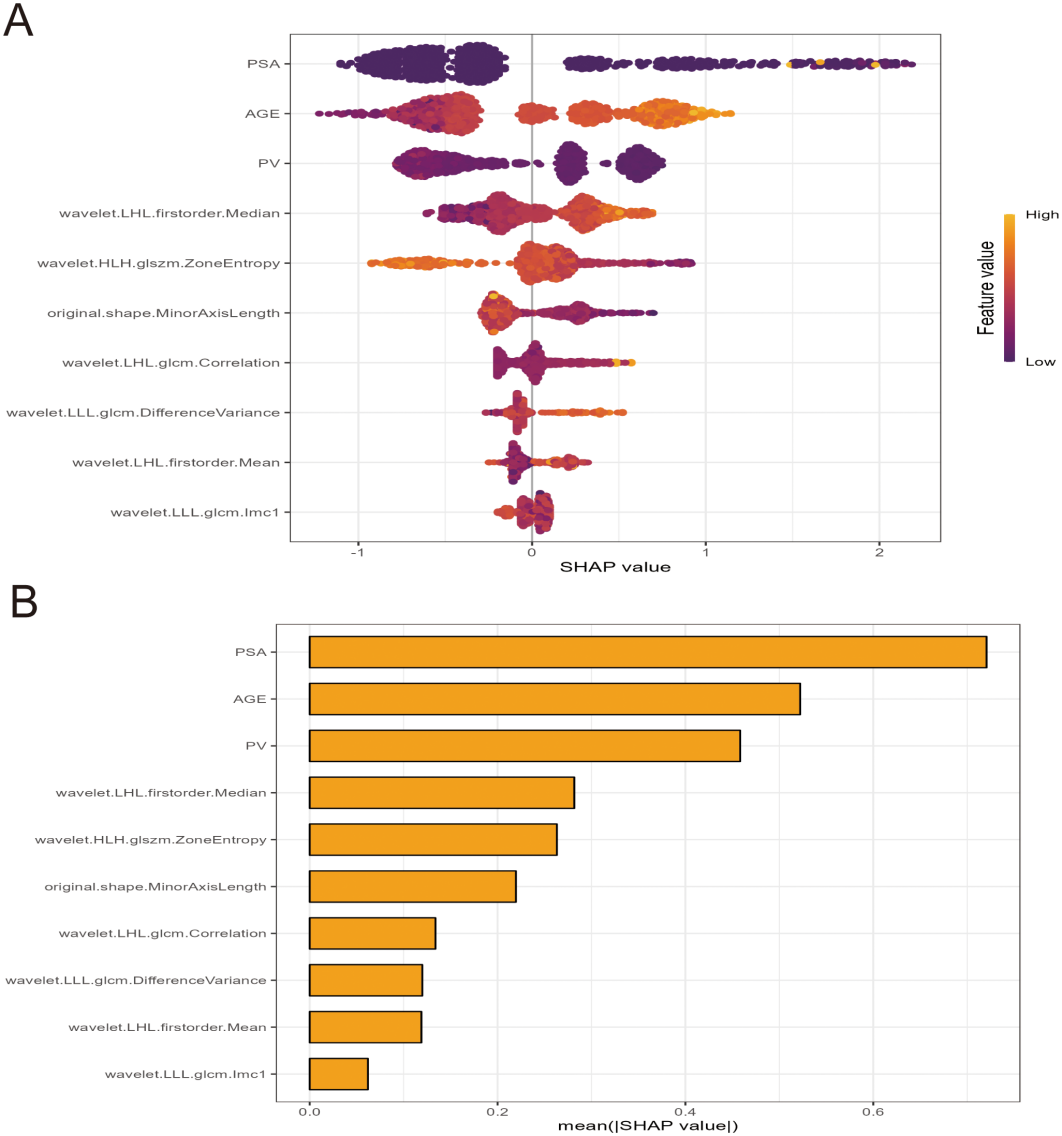
SHAP summary plots for the XGBoost model. **(A)** Beeswarm plot and **(B)** bar plot illustrations of feature relevance and combined feature attributions associated with model predictive performance. SHAP, Shapley additive explanations; XGB, extreme gradient boosting.

To enhance the interpretability of the machine learning models, SHAP analysis was employed to visualize the contribution of individual features in the XGBoost model. **Figure 8** provides the SHAP summary plot, ranking features by their overall impact on the prediction outcomes. The results indicate that PSA, age, prostate volume (PV), wavelet.LHL.firstorder.Median, wavelet.HLH.glszm.ZoneEntropy, and original.shape.MinorAxisLength were the six most influential features in the XGBoost model.

### 3.5 Calibration curves, decision curve analyses, and brier scores

**Figure 9:**
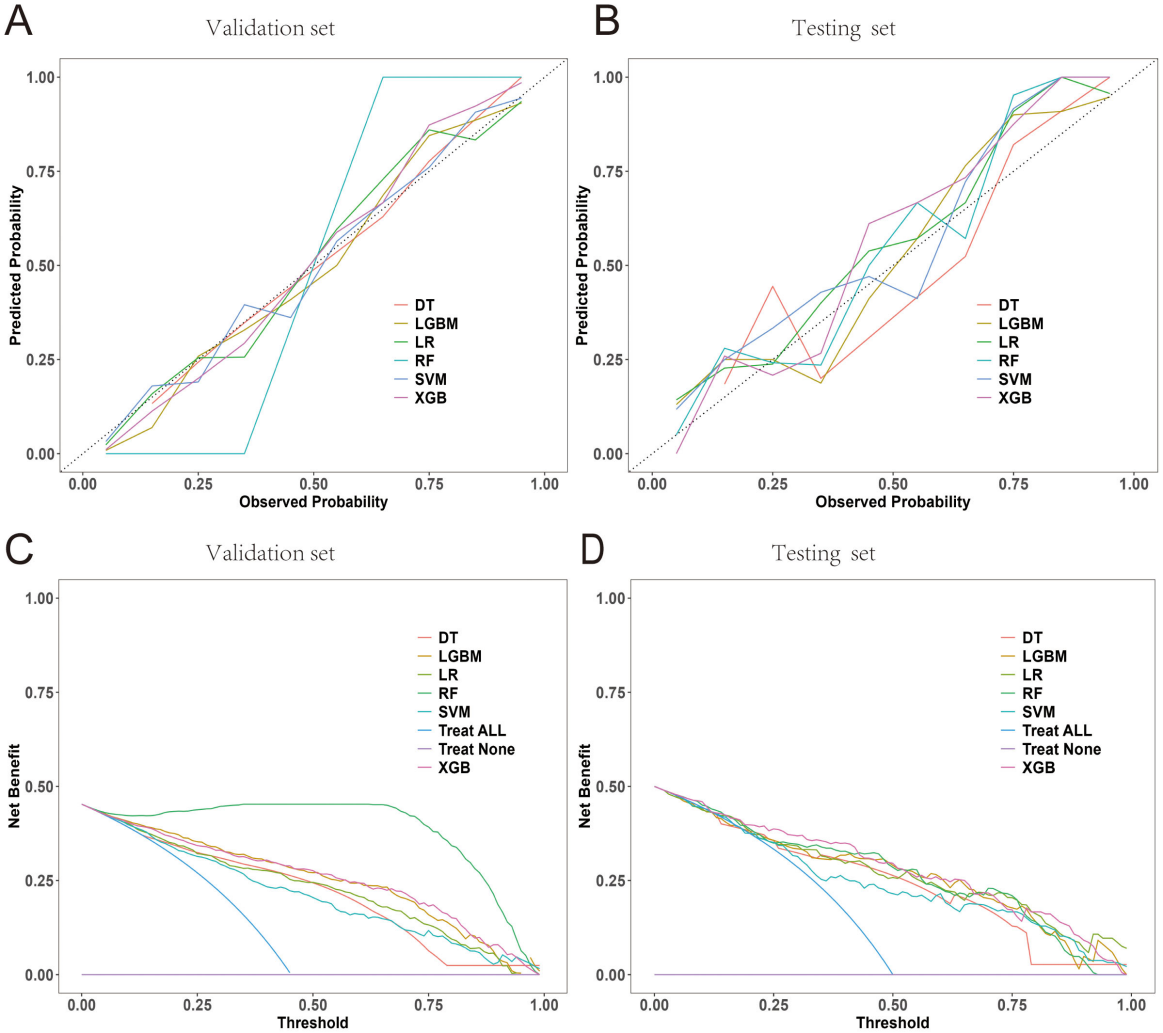
Calibration curves and decision curve. **(A, B)** Calibration curves of the six models for predicting PCa in the internal validation and test cohorts. **(C, D)** DCA for the six models in the internal validation and test cohorts.

Calibration curves for the six models were constructed in both the testing and validation datasets to evaluate the concordance between the predicted PCA probability and the observed outcomes (**Figures 9A, B**). As shown in the Figure, the bias curves closely approximated the ideal line, indicating a robust agreement between the predictions of the model and the observed results. Subsequent DCA results demonstrated that the six models offered superior net benefits for predicting PCa compared to the “treat all or none” strategy across most of the risk thresholds (**Figures 8C, D**). Additionally, as shown in **Table 5**, the Brier scores of the six models were determined to evaluate the accuracy of the probability predictions, indicating superior performance by the XGBoost model.

**Table 5:** Brier scores of six models.

No.	Model	Testing set	Validation set
1	xgboost	0.1439 (0.1181 - 0.1738)	0.1295 (0.1175 - 0.1423)
2	SVM	0.1828 (0.1551 - 0.2133)	0.1687 (0.1559 - 0.1827)
3	RF	0.1537 (0.1281 - 0.1811)	0.0224 (0.0207 - 0.0247)
4	LR	0.1615 (0.1346 - 0.1971)	0.1515 (0.1383 - 0.1659)
5	lgbm	0.1597 (0.1320 - 0.1935)	0.1287 (0.1165 - 0.1431)
6	DT	0.1734 (0.1410 - 0.2098)	0.1588 (0.1418 - 0.1745)

## 4 Discussion

Integrating radiomics and machine learning(ML) offers immense value and is a significant advancement in the field of medical diagnostics. Here, seven radiomic features were successfully extracted from TRUS video clips using LASSO regression, and these features were used in combination with three clinical predictors (identified through univariate and multivariate analyses), to develop ML-based predictive models for prostate cancer. Six ML algorithms were implemented, with XGBoost offering the best predictive performance (test AUC = 0.881), outperforming logistic regression (LR), decision trees (DT), random forests (RF), support vector machines (SVM), and LightGBM (LGBM). SHAP analysis provided a greater degree of model interpretability by quantifying feature contributions, overcoming the “black-box” nature that often hampers the use of ML models.

The SHAP analysis revealed that age was significantly associated with prostate cancer incidence. Previous research has shown a link between age and prostate cancer incidence, with older age associated with higher incidence rates. Men over the age of 50 years represent a high-risk group, and over 70% of patients with prostate cancer in the USA are over 65 years old. Similarly, the incidence rate of prostate cancer in China also increases with age.

The results identified age, PV, and tPSA as key predictors using multivariate analysis, while PSAD and f/tPSA did not show significant associations with PCa in the multivariate model (PSAD: p=0.844; f/tPSA: p=0.319). Despite the clinical relevance of PSAD and f/tPSA, as shown by the results of the multivariate logistic regression (**Table 2**), to avoid the incorporation of redundant or non-predictive features that make the model overly complex, only variables with p<0.05 that were retained in the multivariate analysis were included. Therefore, PSAD and f/tPSA were excluded from the final model.

Notably, we conducted a supplementary analysis that included both PSAD and f/tPSA in the XGBoost model. The results showed that the AUC was 0.867 (0.881 in the original model) with an increase in the Brier score (0.1488 vs. 0.1439), indicating that the inclusion did not improve the model performance. This indicates that the predictive value of PSAD and f/tPSA does not increase significantly when combined with selected radiological features, age, PV, and tPSA. Furthermore, from the perspective of biological principles, tPSA and PV already cover the diagnostic information of PSAD.

TRUS and MRI are commonly used imaging modalities for the assessment of prostate disorders. Although MRI does not involve radiation exposure, it is expensive. According to the medical equipment procurement data of our institution, the average cost of acquiring MRI equipment is approximately 15 times that of TRUS, with a single examination costing about eight times more; additionally, MRI also requires specialized technicians and dedicated facility support. In contrast, TRUS equipment is simple to operate and the procedure can be performed rapidly in outpatient clinics, significantly reducing both patient waiting times and medical resource utilization. Furthermore, for early screening of prostate cancer, the greater cost-effectiveness of TRUS renders it more suitable as an initial screening tool, leading to its wide application in clinical practice (14, 15). This approach reduces the risk of missed diagnoses while avoiding medical resource wastage caused by over-reliance on MRI. However, TRUS has limitations in PCa detection. For example, it suffers from issues with central zone lesion identification, as over 70% of PCa cases originate in the peripheral zone, presenting as hypoechoic lesions that are readily detectable on TRUS. However, central zone tumors often blend with the hypoechoic background of the inner gland, leading to missed diagnoses (16, 17). It is also constrained by moderate diagnostic accuracy, as TRUS exhibits a pooled sensitivity of 68% and specificity of 72% in population-based studies (18), highlighting its limitations in early-stage PCa screening.

These challenges underscore the crucial need to develop an advanced approach to feature extraction capable of enhancing TRUS-based diagnostic capabilities. In this study, LASSO regression was used to identify key predictive features, effectively reducing dimensionality while maintaining informative predictors. LASSO is a linear regression technique that eliminates less significant variables by shrinking their coefficients to zero, ensuring that only the most relevant features contribute to the prediction model (19). This approach was employed here to ensure selection of the most relevant and valid features for the predictive model.

The seven valid radiomic features identified in this study reflect the biological characteristics of prostate cancer in different ways (20–22). (1) Morphological features (e.g. MinorAxisLength), which reflect the shape and size of the tumor, and are related to tumor growth patterns and invasiveness. (2) Signal strength (mean/median), which provides a reflection of the overall grayscale characteristics and concentration trends of the tumor, and is related to tumor cell densities, structural uniformity of the tissue, and the heterogeneity of tumor cells. (3) Texture features (correlation/DifferenceVariance/Imc1), which indicate the spatial correlations of grayscale values, which are in turn related to the order of cell arrangement. These are sensitive to changes in the internal structure of the tumor, and provide a reflection of the overall characteristics of the tumor, such as tumor morphology, size, and edge, and assist in determining the degree of malignancy. (4) Heterogeneity (Zone Entropy), a parameter that indicates the complexity and randomness of different gray areas in the image, and provides a reflection of tumor cell diversity and structural complexity of the tissue. In clinical practice, these characteristics can offer valuable information for the diagnosis, treatment, and prognostic prediction of prostate cancer. However, they usually require a comprehensive analysis of clinical data and the results of other examinations.(5) Wavelet features: Wavelet transformation deconstructs the grayscale signals of ultrasound images at multiple scales, enabling capture of microstructural heterogeneity that is difficult to identify in traditional visual assessment; this heterogeneity is closely related to the pathological characteristics of prostate cancer. There are several explanations for the high predictability of wavelet features in PCa diagnosis. First, wavelet features can capture sub-visual pathological changes, detecting microscopic structural abnormalities that are not distinguishable by the naked eye and are more sensitive to changes in early-stage cancer. Second, they are not influenced significantly by image noise and artifacts. The multi-scale deconstruction properties of wavelet transformation can filter out noise while retaining key signals, resulting in better stability. Third, they are associated with the biological behavior of tumors, are correlated with biological indicators such as tumor microvessel density, and can indirectly reflect the invasiveness of cancer cells. Moreover, they show a significant association with “extracapsular extension” in postoperative pathology, thus possessing clear biological significance.

Numerous studies have investigated ML-based predictive models for PCa. Wang et al. (23) applied ML techniques to analyze TRUS video clips, demonstrating that an SVM model outperformed senior radiologists (SRs, with over 10 years of experience) using MRI. The AUCs for their SVM model were 0.78 and 0.75 in the validation and test sets, respectively, demonstrating good diagnostic performance. However, the present study demonstrated superior diagnostic efficacy, with the SVM model achieving AUCs of 0.825 and 0.800 in the validation and test sets, respectively. Specifically, among the six models evaluated, XGBoost exhibited the strongest predictive capability.

XGBoost outperformed SVM across three critical dimensions (8, 24, 25): (1) Predictive accuracy: As an ensemble learning algorithm, XGBoost combines multiple weak learners to reduce bias, handle complex data structures, and manage missing values, thereby enhancing predictive accuracy and data utilization. SVM, while effective in processing linear data, struggles with complex datasets due to challenges in selecting appropriate kernel functions, making its predictive accuracy highly sensitive to kernel selection; (2) Computational efficiency: XGBoost supports parallel processing, enabling efficient computation on multi-core CPUs. Its feature-splitting mechanism during tree construction ensures computational independence, reducing training time for large datasets. In contrast, SVM incurs significant computational costs when handling high-dimensional data, as the complexity of kernel function calculations increases exponentially with data scale; (3) Model flexibility: XGBoost employs an intuitive decision tree-based structure, allowing for direct calculation of feature importance and flexible parameter tuning. Conversely, once an SVM model’s kernel function is determined, its structure remains relatively rigid, limiting adaptability.

Additionally, several studies have explored PSA-based PCa prediction models. For example, Shi et al. (26) analyzed clinical data from MRI-negative patients who underwent prostate biopsy, identifying age, PSA, PSAD, and PV as key predictors. Their logistic regression-based model achieved an AUC of 0.774, which was lower than the predictive performance of the present ML-based approach. Their study relied solely on clinical data and employed a single modeling technique (logistic regression), whereas this study leveraged multiple ML algorithms to enhance predictive accuracy, potentially yielding a more clinically meaningful model.

Currently, most clinical diagnostic approaches rely on isolated biomarkers such as PSA and traditional imaging assessments for PCa prediction. The present study demonstrates that integrating clinical parameters with radiomics-based feature extraction substantially improves diagnostic performance. Radiomics, a cutting-edge analytical technique, enables comprehensive disease characterization by extracting intricate quantitative features from medical images, vastly outperforming the diagnostic potential of conventional clinical data alone. Consequently, the integrated model—incorporating PSA, additional biochemical markers, and radiomic features—developed herein was able to achieve superior predictive accuracy compared to models based solely on PSA or imaging data. This approach not only affords enhanced clinical decision-making but also provides more precise diagnostic and treatment strategies, ultimately improving patient outcomes and prognosis.

In recent years, nomograms, which integrate multiple risk factors, have become widely used in the prediction of medical prognosis and outcomes, offering a clearer, more concise, and easily comprehensible approach (9, 27). Zhang et al. (21) developed a nomogram by combining MRI-based radiomics scores with PI-RADS V2.1 classification and age. This achieved an AUC value of 0.953 in the validation set, demonstrating excellent calibration and clinical utility, which could reduce the performance of unnecessary prostate biopsies in patients with PSA values in the gray zone. Its predictive performance surpassed that of this study (XGBoost model AUC: 0.881). This may be attributed to the typical provision of higher-resolution images by MRI, as these are capable of capturing more anatomical details, particularly excelling in soft-tissue contrast imaging compared to ultrasound. This suggests that MRI-based data may include more discriminative features, which are crucial for model performance.

The present study employed six machine-learning models, including a logistic regression model (test set AUC, 0.843). The nomogram is a visualization tool based on logistic regression models, and while it has significant advantages, such as simple structure, effective visualization, and interpretability, it may have limited capability in the handling of complex data. The results showed superior performance of the XGBoost model (test set AUC, 0.881). Therefore, we chose to build the model using XGBoost combined with the visualization tool SHAP for interpretation, improving the interpretability and replacing the functionality of traditional nomograms.

The core advantage of XGBoost compared to nomograms lies in its powerful predictive performance and flexibility, as it can handle it high-dimensional, nonlinear data and complex interaction relationships efficiently through a gradient-boosting framework, which supports the selection of automatic features, ensures regularization to prevent overfitting, and can process missing values directly. It is suitable for various tasks such as classification and regression, excelling particularly in the analysis of large-scale data and complex scenarios.

In this study, the AUC value of the XGBoost model was found to be 0.881, indicating excellent discriminative ability. However, its Brier score was 0.1439, while the calibration error was classified as medium. Nevertheless, there is still room for improved calibration. The inherent complexity of the data and the characteristics of the tree structure model may have contributed to calibration errors. As shown in **Figure 9**, its calibration curve is essentially distributed along the ideal diagonal. The predicted probability is relatively close to the actual probability, although there is a slight overestimation in the low-probability area (<0.2) and a slight underestimation in the high-probability area (>0.8), maintaining the Brier score at a medium level. This moderate calibration error may affect the practical application of the model, especially in clinical settings where accurate probability estimation is crucial for decision-making. In our future work, we plan to explore calibration techniques, such as Platt scaling or isobaric regression, to improve the calibration of the XGBoost model, thereby reducing the Brier score and enhancing the clinical practicality of the model.

### 4.1 Limitations

Despite its promising results, this study has several limitations.

For one, as a retrospective study, these findings may be influenced by inherent biases. However, the inclusion of 928 patients enhances the reliability of these results. Future prospective validation studies are required to corroborate these findings.

Secondly, the study was conducted at a single center and is thus prone to selection bias (28). Despite random splitting of the training and test cohorts, the sample source was relatively limited and the findings may suffer from a lack of generalizability to other regions or populations. These issues will be addressed in future studies. Specifically, the data source will be expanded to include not only Dongyang Hospital of Wenzhou Medical University but a new data source (Zhejiang Cancer Hospital) to increase the diversity and representativeness of the samples. Multi-center external validation studies (29, 30) will also be conducted in the future. Secondly, methods such as propensity scores (31) will be adopted for data preprocessing to balance the features between the groups and reduce the influence of selection bias. Furthermore, during the research design stage, we will establish stricter inclusion and exclusion criteria to ensure the homogeneity of the sample and will also conduct sensitivity analysis to evaluate the robustness of the results.

Thirdly, familial inheritance is a further significant factor (32, 33), accounting for approximately 10% of prostate cancer cases. Men with a family history of specific cancer syndromes face a higher risk of prostate cancer. This study did not include data on family history but these will be included in future studies to refine the model. We fully recognize the importance of these variables for the generalizability of the model. Subsequent studies will optimize the design to ensure comprehensive collection of relevant information, thereby further validating the effectiveness of the model across diverse populations.

In addition, when obtaining videos, the probe should theoretically move at a constant speed. However, manual operation has difficulty in matching the accuracy of machines.

Lastly, this study incorporated dynamic TRUS video analysis, but additional imaging techniques such as contrast-enhanced ultrasound and shear wave elastography were not included. Future studies integrating these modalities into ML models may further enhance diagnostic performance.

## 5 Conclusion

Based on these results, machine learning models based on LR, DT, SVM, RF, XGBoost, and LightGBM models demonstrate significant diagnostic utility for the non-invasive early detection of prostate cancer. Notably, the XGBoost model outperformed all other models in terms of its predictive performance. These models have practical applications in clinical decision-making, and may be able to assist physicians in early diagnosis and treatment planning for prostate cancer patients. Moreover, they can provide valuable guidance for prostate biopsy procedures and patient follow-up, ultimately improving clinical outcomes.

## Data Availability

The data analyzed in this study is subject to the following licenses/restrictions: The data analyzed in the current study are available from the corresponding author upon reasonable request. Requests to access these datasets should be directed to duyh520@outlook.com.
