# Supplementary figures and images for: Evaluation of a PSA and transrectal prostate ultrasound video-based machine learning model as a tool for prostate cancer diagnosis
Source: Front Oncol. 2025 Sep 8;15:1590396. doi: 10.3389/fonc.2025.1590396 (PMC12450705; doi:10.3389/fonc.2025.1590396)

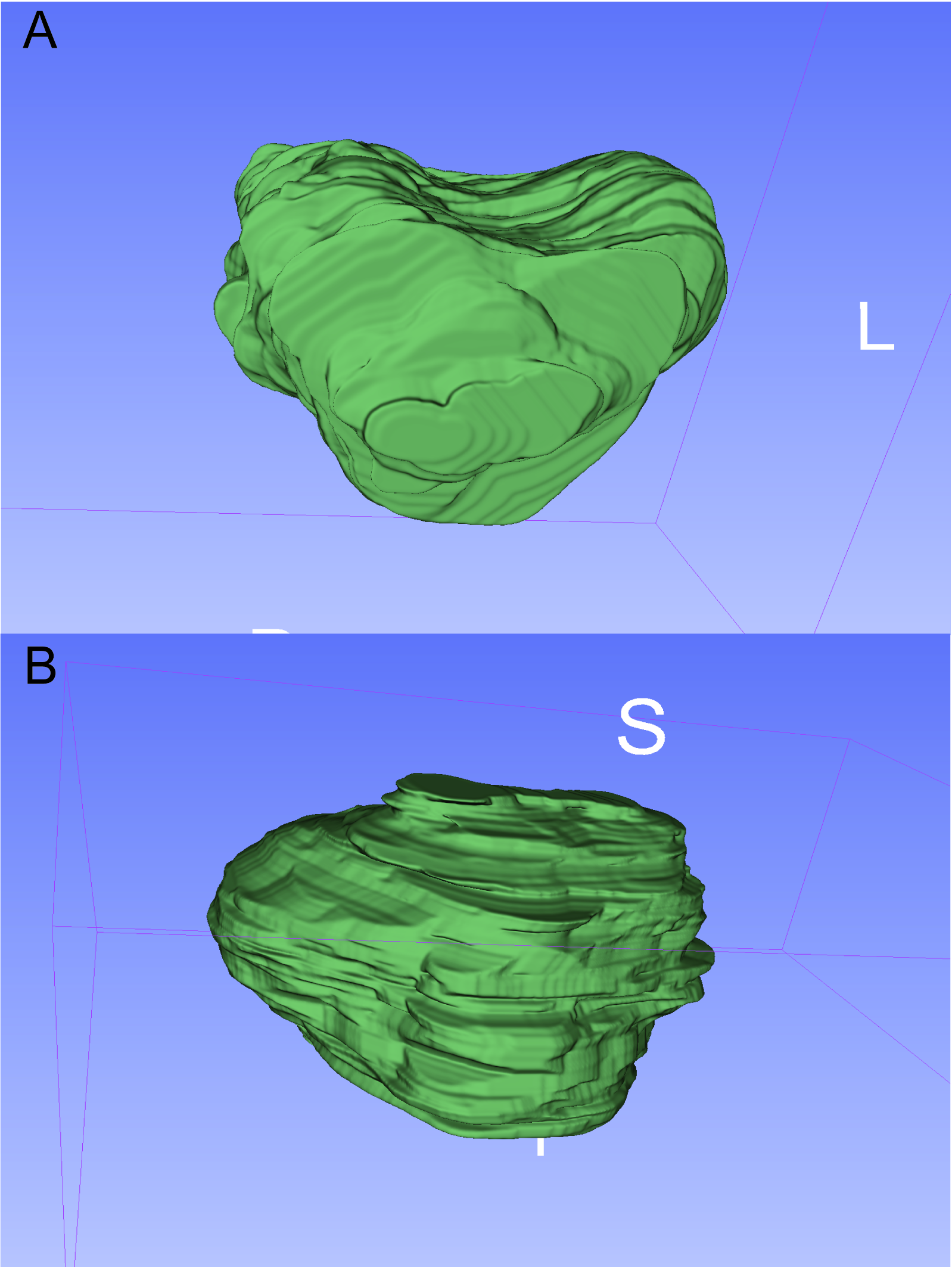

Supplement: Supplementary file 1 [file Image1.tif]
