# Supplementary material for: Evaluation of a PSA and transrectal prostate ultrasound video-based machine learning model as a tool for prostate cancer diagnosis
Source: Front Oncol. 2025 Sep 8;15:1590396. doi: 10.3389/fonc.2025.1590396 (PMC12450705; doi:10.3389/fonc.2025.1590396)
Supplement: Supplementary file 2 [file Table1.docx]

Supplementary Figure S1

3D stereoscopic prostate image.

Supplementary Table S1

| **Feature** | **icc1** | **icc2** |
| --- | --- | --- |
| original.shape.LeastAxisLength | 0.987647286 | 0.996781174 |
| original.shape.MajorAxisLength | 0.993186832 | 0.994944424 |
| original.shape.Maximum2DDiameterColumn | 0.992058023 | 0.889661107 |
| original.shape.Maximum2DDiameterRow | 0.961073284 | 0.886637985 |
| original.shape.Maximum2DDiameterSlice | 0.951297612 | 0.902775717 |
| original.shape.Maximum3DDiameter | 0.984635403 | 0.914188214 |
| original.shape.MeshVolume | 0.975367534 | 0.907751073 |
| original.shape.MinorAxisLength | 0.992624304 | 0.998692404 |
| original.shape.Sphericity | 0.94389769 | 0.932426689 |
| original.shape.SurfaceArea | 0.834431934 | 0.908403506 |
| original.shape.SurfaceVolumeRatio | 0.981731175 | 0.986889096 |
| original.shape.VoxelVolume | 0.907510327 | 0.953094345 |
| original.firstorder.10Percentile | 0.992628537 | 0.998697315 |
| original.firstorder.90Percentile | 0.997546012 | 0.996857454 |
| original.firstorder.Energy | 0.987115193 | 0.985005089 |
| original.firstorder.Entropy | 0.987610161 | 0.99717937 |
| original.firstorder.InterquartileRange | 0.948255451 | 0.971310874 |
| original.firstorder.Kurtosis | 0.937188554 | 0.958410358 |
| original.firstorder.Maximum | 0.826179753 | 0.980744551 |
| original.firstorder.Mean | 0.913633164 | 0.948717384 |
| original.firstorder.Median | 0.995791559 | 0.991273419 |
| original.firstorder.Minimum | 0.997446434 | 0.992576848 |
| original.firstorder.Range | 1 | 1 |
| original.firstorder.RobustMeanAbsoluteDeviation | 0.885569856 | 0.988378084 |
| original.firstorder.RootMeanSquared | 0.957217028 | 0.955596199 |
| original.firstorder.Skewness | 0.99306113 | 0.989074024 |
| original.firstorder.TotalEnergy | 0.945324708 | 0.994781468 |
| original.firstorder.Uniformity | 0.987610161 | 0.99717937 |
| original.firstorder.Variance | 0.967468556 | 0.976259902 |
| original.glcm.Autocorrelation | 0.865001669 | 0.935911562 |
| original.glcm.ClusterProminence | 0.995191837 | 0.983822402 |
| original.glcm.ClusterShade | 0.752628844 | 0.930191152 |
| original.glcm.ClusterTendency | 0.874971808 | 0.981217687 |
| original.glcm.Contrast | 0.904943484 | 0.949985125 |
| original.glcm.Correlation | 0.984367091 | 0.968181002 |
| original.glcm.DifferenceAverage | 0.949399374 | 0.986684647 |
| original.glcm.DifferenceEntropy | 0.990986876 | 0.986166083 |
| original.glcm.DifferenceVariance | 0.985254065 | 0.980754034 |
| original.glcm.Id | 0.967007068 | 0.945814045 |
| original.glcm.Idm | 0.992762318 | 0.993814667 |
| original.glcm.Idmn | 0.992837952 | 0.99275971 |
| original.glcm.Idn | 0.901926141 | 0.912349105 |
| original.glcm.Imc1 | 0.942971377 | 0.957402807 |
| original.glcm.Imc2 | 0.965330578 | 0.993149734 |
| original.glcm.InverseVariance | 0.960586348 | 0.983873695 |
| original.glcm.JointAverage | 0.984483951 | 0.994356995 |
| original.glcm.JointEnergy | 0.99690624 | 0.990512144 |
| original.glcm.JointEntropy | 0.987546849 | 0.991551498 |
| original.glcm.MaximumProbability | 0.954387564 | 0.966177336 |
| original.glcm.MCC | 0.980423242 | 0.980626908 |
| original.glcm.SumAverage | 0.985435221 | 0.980686824 |
| original.glcm.SumEntropy | 0.99694704 | 0.991389117 |
| original.glcm.SumSquares | 0.964317451 | 0.973807999 |
| original.gldm.DependenceEntropy | 0.90717559 | 0.955183463 |
| original.gldm.DependenceNonUniformity | 0.943538194 | 0.969999028 |
| original.gldm.DependenceNonUniformityNormalized | 0.992985326 | 0.998843934 |
| original.gldm.DependenceVariance | 0.99527062 | 0.992254814 |
| original.gldm.GrayLevelNonUniformity | 0.991835854 | 0.997094279 |
| original.gldm.GrayLevelVariance | 0.993623823 | 0.996438014 |
| original.gldm.HighGrayLevelEmphasis | 0.874722809 | 0.942777871 |
| original.gldm.LargeDependenceEmphasis | 0.992895249 | 0.983267123 |
| original.gldm.LargeDependenceHighGrayLevelEmphasis | 0.990707356 | 0.997033529 |
| original.gldm.LargeDependenceLowGrayLevelEmphasis | 0.997854065 | 0.992324092 |
| original.gldm.LowGrayLevelEmphasis | 0.986340595 | 0.999158428 |
| original.gldm.SmallDependenceEmphasis | 0.99389664 | 0.999153754 |
| original.gldm.SmallDependenceHighGrayLevelEmphasis | 0.982573928 | 0.995032887 |
| original.gldm.SmallDependenceLowGrayLevelEmphasis | 0.987927502 | 0.994201446 |
| original.glrlm.GrayLevelNonUniformity | 0.984952987 | 0.995085295 |
| original.glrlm.GrayLevelNonUniformityNormalized | 0.994547328 | 0.997155415 |
| original.glrlm.GrayLevelVariance | 0.939229163 | 0.971075944 |
| original.glrlm.HighGrayLevelRunEmphasis | 0.804832115 | 0.931771222 |
| original.glrlm.LongRunEmphasis | 0.989064277 | 0.981736823 |
| original.glrlm.LongRunHighGrayLevelEmphasis | 0.981912537 | 0.998162637 |
| original.glrlm.LongRunLowGrayLevelEmphasis | 0.992898493 | 0.985257536 |
| original.glrlm.LowGrayLevelRunEmphasis | 0.97998159 | 0.99880189 |
| original.glrlm.RunEntropy | 0.996637637 | 0.998741998 |
| original.glrlm.RunLengthNonUniformity | 0.86121192 | 0.968397497 |
| original.glrlm.RunLengthNonUniformityNormalized | 0.992387603 | 0.998794727 |
| original.glrlm.RunPercentage | 0.992421913 | 0.994066928 |
| original.glrlm.RunVariance | 0.991847352 | 0.995716788 |
| original.glrlm.ShortRunEmphasis | 0.978981333 | 0.998191496 |
| original.glrlm.ShortRunHighGrayLevelEmphasis | 0.991862373 | 0.995196109 |
| original.glrlm.ShortRunLowGrayLevelEmphasis | 0.986972807 | 0.980673445 |
| original.glszm.GrayLevelNonUniformity | 0.99746234 | 0.998317526 |
| original.glszm.GrayLevelNonUniformityNormalized | 0.994393197 | 0.994349249 |
| original.glszm.GrayLevelVariance | 0.813999546 | 0.935983974 |
| original.glszm.HighGrayLevelZoneEmphasis | 0.836218296 | 0.986013137 |
| original.glszm.LargeAreaEmphasis | 0.944384105 | 0.988940783 |
| original.glszm.LargeAreaHighGrayLevelEmphasis | 0.980243587 | 0.995622475 |
| original.glszm.LargeAreaLowGrayLevelEmphasis | 0.987372839 | 0.996920363 |
| original.glszm.LowGrayLevelZoneEmphasis | 0.951526304 | 0.997308258 |
| original.glszm.SizeZoneNonUniformity | 0.989915479 | 0.996545804 |
| original.glszm.SizeZoneNonUniformityNormalized | 0.997227214 | 0.995619043 |
| original.glszm.SmallAreaEmphasis | 0.870351894 | 0.802075047 |
| original.glszm.SmallAreaHighGrayLevelEmphasis | 0.801335158 | 0.843626998 |
| original.glszm.SmallAreaLowGrayLevelEmphasis | 0.975820378 | 0.997469594 |
| original.glszm.ZoneEntropy | 0.991071454 | 0.994349672 |
| original.glszm.ZoneVariance | 0.940334224 | 0.977800379 |
| original.ngtdm.Busyness | 0.980289317 | 0.995627076 |
| original.ngtdm.Coarseness | 0.974133258 | 0.981849518 |
| original.ngtdm.Complexity | 0.994736148 | 0.995915582 |
| original.ngtdm.Contrast | 0.988997028 | 0.7977014 |
| original.ngtdm.Strength | 0.934025357 | 0.911889371 |
| wavelet.LLH.firstorder.10Percentile | 0.865676215 | 0.888425999 |
| wavelet.LLH.firstorder.90Percentile | 0.988125441 | 0.997533208 |
| wavelet.LLH.firstorder.Energy | 0.979857832 | 0.998041306 |
| wavelet.LLH.firstorder.Entropy | 0.963519868 | 0.997984536 |
| wavelet.LLH.firstorder.InterquartileRange | 0.967967418 | 0.998923741 |
| wavelet.LLH.firstorder.Kurtosis | 0.988781279 | 0.997812758 |
| wavelet.LLH.firstorder.Mean | 0.97439005 | 0.998560659 |
| wavelet.LLH.firstorder.Median | 0.772301646 | 0.9513549 |
| wavelet.LLH.firstorder.Minimum | 0.958456907 | 0.965500176 |
| wavelet.LLH.firstorder.Range | 0.983802086 | 0.913266569 |
| wavelet.LLH.firstorder.RobustMeanAbsoluteDeviation | 0.771868976 | 0.916596636 |
| wavelet.LLH.firstorder.RootMeanSquared | 0.987807871 | 0.997880799 |
| wavelet.LLH.firstorder.Skewness | 0.916944599 | 0.998912548 |
| wavelet.LLH.firstorder.TotalEnergy | 0.820658234 | 0.85384473 |
| wavelet.LLH.firstorder.Uniformity | 0.963519868 | 0.997984536 |
| wavelet.LLH.firstorder.Variance | 0.982326695 | 0.998820768 |
| wavelet.LLH.glcm.Autocorrelation | 0.922274835 | 0.998868044 |
| wavelet.LLH.glcm.ClusterProminence | 0.978059315 | 0.930039688 |
| wavelet.LLH.glcm.ClusterShade | 0.805356603 | 0.896348952 |
| wavelet.LLH.glcm.ClusterTendency | 0.839262234 | 0.950986627 |
| wavelet.LLH.glcm.Contrast | 0.943352462 | 0.996935706 |
| wavelet.LLH.glcm.Correlation | 0.958374053 | 0.998927107 |
| wavelet.LLH.glcm.DifferenceAverage | 0.975054509 | 0.931561299 |
| wavelet.LLH.glcm.DifferenceEntropy | 0.981623611 | 0.998917379 |
| wavelet.LLH.glcm.DifferenceVariance | 0.9742312 | 0.999069528 |
| wavelet.LLH.glcm.Id | 0.904766493 | 0.99894752 |
| wavelet.LLH.glcm.Idm | 0.988105101 | 0.998990121 |
| wavelet.LLH.glcm.Idmn | 0.988258111 | 0.998947837 |
| wavelet.LLH.glcm.Idn | 0.941810696 | 0.849615207 |
| wavelet.LLH.glcm.Imc1 | 0.900916069 | 0.889381917 |
| wavelet.LLH.glcm.Imc2 | 0.92197906 | 0.998913407 |
| wavelet.LLH.glcm.InverseVariance | 0.933860176 | 0.997623453 |
| wavelet.LLH.glcm.JointAverage | 0.979508291 | 0.996870606 |
| wavelet.LLH.glcm.JointEnergy | 0.973419633 | 0.922769256 |
| wavelet.LLH.glcm.JointEntropy | 0.984990124 | 0.997754398 |
| wavelet.LLH.glcm.MaximumProbability | 0.962329703 | 0.946667308 |
| wavelet.LLH.glcm.MCC | 0.979240386 | 0.997753434 |
| wavelet.LLH.glcm.SumAverage | 0.990550884 | 0.997862236 |
| wavelet.LLH.glcm.SumEntropy | 0.973485448 | 0.922933518 |
| wavelet.LLH.glcm.SumSquares | 0.973536478 | 0.995714526 |
| wavelet.LLH.gldm.DependenceEntropy | 0.975953321 | 0.999023601 |
| wavelet.LLH.gldm.DependenceNonUniformity | 0.95819234 | 0.997434661 |
| wavelet.LLH.gldm.DependenceNonUniformityNormalized | 0.993238658 | 0.998638719 |
| wavelet.LLH.gldm.DependenceVariance | 0.998590185 | 0.992242656 |
| wavelet.LLH.gldm.GrayLevelNonUniformity | 0.997739856 | 0.988999466 |
| wavelet.LLH.gldm.GrayLevelVariance | 0.994270123 | 0.998908182 |
| wavelet.LLH.gldm.HighGrayLevelEmphasis | 0.922149244 | 0.998882306 |
| wavelet.LLH.gldm.LargeDependenceEmphasis | 0.978010921 | 0.930938525 |
| wavelet.LLH.gldm.LargeDependenceHighGrayLevelEmphasis | 0.987322639 | 0.998934187 |
| wavelet.LLH.gldm.LargeDependenceLowGrayLevelEmphasis | 0.982683566 | 0.93877776 |
| wavelet.LLH.gldm.LowGrayLevelEmphasis | 0.962722816 | 0.948574601 |
| wavelet.LLH.gldm.SmallDependenceEmphasis | 0.964311719 | 0.906251175 |
| wavelet.LLH.gldm.SmallDependenceHighGrayLevelEmphasis | 0.976608873 | 0.99168486 |
| wavelet.LLH.gldm.SmallDependenceLowGrayLevelEmphasis | 0.976406563 | 0.926612506 |
| wavelet.LLH.glrlm.GrayLevelNonUniformity | 0.972687691 | 0.850603282 |
| wavelet.LLH.glrlm.GrayLevelNonUniformityNormalized | 0.994054138 | 0.998794648 |
| wavelet.LLH.glrlm.GrayLevelVariance | 0.97696319 | 0.99892662 |
| wavelet.LLH.glrlm.HighGrayLevelRunEmphasis | 0.892533709 | 0.998893694 |
| wavelet.LLH.glrlm.LongRunEmphasis | 0.977736041 | 0.930172604 |
| wavelet.LLH.glrlm.LongRunHighGrayLevelEmphasis | 0.98362504 | 0.999465183 |
| wavelet.LLH.glrlm.LongRunLowGrayLevelEmphasis | 0.984630906 | 0.951221052 |
| wavelet.LLH.glrlm.LowGrayLevelRunEmphasis | 0.954493308 | 0.938950284 |
| wavelet.LLH.glrlm.RunEntropy | 0.963392693 | 0.904167262 |
| wavelet.LLH.glrlm.RunLengthNonUniformity | 0.849015515 | 0.996439002 |
| wavelet.LLH.glrlm.RunLengthNonUniformityNormalized | 0.991037506 | 0.998228285 |
| wavelet.LLH.glrlm.RunPercentage | 0.986330793 | 0.999121776 |
| wavelet.LLH.glrlm.RunVariance | 0.987212652 | 0.999126071 |
| wavelet.LLH.glrlm.ShortRunEmphasis | 0.983593667 | 0.999477567 |
| wavelet.LLH.glrlm.ShortRunHighGrayLevelEmphasis | 0.985905084 | 0.999139971 |
| wavelet.LLH.glrlm.ShortRunLowGrayLevelEmphasis | 0.977674447 | 0.931401831 |
| wavelet.LLH.glszm.GrayLevelNonUniformity | 0.964654598 | 0.890751652 |
| wavelet.LLH.glszm.GrayLevelNonUniformityNormalized | 0.996497035 | 0.998247269 |
| wavelet.LLH.glszm.GrayLevelVariance | 0.974902776 | 0.997970349 |
| wavelet.LLH.glszm.HighGrayLevelZoneEmphasis | 0.850321525 | 0.998624031 |
| wavelet.LLH.glszm.LargeAreaEmphasis | 0.97578743 | 0.925715705 |
| wavelet.LLH.glszm.LargeAreaHighGrayLevelEmphasis | 0.992745107 | 0.998573085 |
| wavelet.LLH.glszm.LargeAreaLowGrayLevelEmphasis | 0.992188838 | 0.979792409 |
| wavelet.LLH.glszm.LowGrayLevelZoneEmphasis | 0.988621737 | 0.993904562 |
| wavelet.LLH.glszm.SizeZoneNonUniformity | 0.956026915 | 0.872826689 |
| wavelet.LLH.glszm.SizeZoneNonUniformityNormalized | 0.996625496 | 0.996438783 |
| wavelet.LLH.glszm.SmallAreaEmphasis | 0.9900416 | 0.967846179 |
| wavelet.LLH.glszm.SmallAreaHighGrayLevelEmphasis | 0.986832903 | 0.962825285 |
| wavelet.LLH.glszm.SmallAreaLowGrayLevelEmphasis | 0.975080763 | 0.906776045 |
| wavelet.LLH.glszm.ZoneEntropy | 0.961882059 | 0.879234321 |
| wavelet.LLH.glszm.ZonePercentage | 0.953277062 | 0.997560257 |
| wavelet.LLH.glszm.ZoneVariance | 0.944268931 | 0.972325901 |
| wavelet.LLH.ngtdm.Busyness | 0.992748624 | 0.998573698 |
| wavelet.LLH.ngtdm.Coarseness | 0.991443024 | 0.936270735 |
| wavelet.LLH.ngtdm.Complexity | 0.988387683 | 0.996995995 |
| wavelet.LLH.ngtdm.Contrast | 0.774386059 | 0.958986561 |
| wavelet.LLH.ngtdm.Strength | 0.968509847 | 0.880834865 |
| wavelet.LHL.firstorder.10Percentile | 0.864295521 | 0.971213886 |
| wavelet.LHL.firstorder.90Percentile | 0.990689988 | 0.912654951 |
| wavelet.LHL.firstorder.Energy | 0.995435898 | 0.943470743 |
| wavelet.LHL.firstorder.Entropy | 0.985836554 | 0.984077668 |
| wavelet.LHL.firstorder.InterquartileRange | 0.990297363 | 0.949060525 |
| wavelet.LHL.firstorder.Kurtosis | 0.995073657 | 0.926397812 |
| wavelet.LHL.firstorder.Maximum | 0.948810688 | 0.99682208 |
| wavelet.LHL.firstorder.Mean | 0.99051232 | 0.924157535 |
| wavelet.LHL.firstorder.Median | 0.949730216 | 0.979708984 |
| wavelet.LHL.firstorder.Minimum | 0.959826273 | 0.989112201 |
| wavelet.LHL.firstorder.RootMeanSquared | 0.995015348 | 0.9278671 |
| wavelet.LHL.firstorder.Skewness | 0.982970318 | 0.920457238 |
| wavelet.LHL.firstorder.TotalEnergy | 0.815527345 | 0.999255106 |
| wavelet.LHL.firstorder.Uniformity | 0.985836554 | 0.984077668 |
| wavelet.LHL.firstorder.Variance | 0.995210853 | 0.966158597 |
| wavelet.LHL.firstorder.Variance | 0.937883257 | 0.824848838 |
| wavelet.LHL.glcm.Autocorrelation | 0.97745359 | 0.869745898 |
| wavelet.LHL.glcm.ClusterProminence | 0.962344823 | 0.915645926 |
| wavelet.LHL.glcm.ClusterShade | 0.80450611 | 0.941022835 |
| wavelet.LHL.glcm.Contrast | 0.974850217 | 0.86184251 |
| wavelet.LHL.glcm.Correlation | 0.982062492 | 0.854250158 |
| wavelet.LHL.glcm.DifferenceAverage | 0.848332465 | 0.984134634 |
| wavelet.LHL.glcm.DifferenceEntropy | 0.992350497 | 0.911229725 |
| wavelet.LHL.glcm.DifferenceVariance | 0.992382241 | 0.94582666 |
| wavelet.LHL.glcm.Id | 0.971241084 | 0.856398448 |
| wavelet.LHL.glcm.Idm | 0.996190323 | 0.94502712 |
| wavelet.LHL.glcm.Idmn | 0.996285587 | 0.944899113 |
| wavelet.LHL.glcm.Idn | 0.828093101 | 0.958949816 |
| wavelet.LHL.glcm.Imc1 | 0.778479301 | 0.82362163 |
| wavelet.LHL.glcm.Imc2 | 0.862419972 | 0.831087343 |
| wavelet.LHL.glcm.InverseVariance | 0.921812968 | 0.893791157 |
| wavelet.LHL.glcm.JointAverage | 0.985665782 | 0.836286005 |
| wavelet.LHL.glcm.JointEnergy | 0.864309977 | 0.823251447 |
| wavelet.LHL.glcm.JointEntropy | 0.997368262 | 0.979537349 |
| wavelet.LHL.glcm.MaximumProbability | 0.753140328 | 0.848454134 |
| wavelet.LHL.glcm.MCC | 0.992476296 | 0.949836641 |
| wavelet.LHL.glcm.SumAverage | 0.997190767 | 0.968513896 |
| wavelet.LHL.glcm.SumEntropy | 0.89913957 | 0.769312988 |
| wavelet.LHL.glcm.SumSquares | 0.990595633 | 0.950731893 |
| wavelet.LHL.gldm.DependenceEntropy | 0.977488025 | 0.848035288 |
| wavelet.LHL.gldm.DependenceNonUniformity | 0.985339828 | 0.962985144 |
| wavelet.LHL.gldm.DependenceNonUniformityNormalized | 0.992102541 | 0.998079787 |
| wavelet.LHL.gldm.DependenceVariance | 0.991706714 | 0.96229937 |
| wavelet.LHL.gldm.GrayLevelNonUniformity | 0.985068813 | 0.761874993 |
| wavelet.LHL.gldm.GrayLevelVariance | 0.993255184 | 0.995240075 |
| wavelet.LHL.gldm.HighGrayLevelEmphasis | 0.977163918 | 0.866040636 |
| wavelet.LHL.gldm.LargeDependenceHighGrayLevelEmphasis | 0.997878747 | 0.95861028 |
| wavelet.LHL.gldm.SmallDependenceEmphasis | 0.993342387 | 0.852791141 |
| wavelet.LHL.gldm.SmallDependenceHighGrayLevelEmphasis | 0.994201299 | 0.92660915 |
| wavelet.LHL.gldm.SmallDependenceLowGrayLevelEmphasis | 0.807107799 | 0.764628809 |
| wavelet.LHL.glrlm.GrayLevelNonUniformityNormalized | 0.993682928 | 0.99689841 |
| wavelet.LHL.glrlm.GrayLevelNonUniformityNormalized | 0.752606187 | 0.894318659 |
| wavelet.LHL.glrlm.GrayLevelVariance | 0.994244827 | 0.97312199 |
| wavelet.LHL.glrlm.HighGrayLevelRunEmphasis | 0.974496319 | 0.880679659 |
| wavelet.LHL.glrlm.LongRunEmphasis | 0.876872913 | 0.88859935 |
| wavelet.LHL.glrlm.LongRunHighGrayLevelEmphasis | 0.997165766 | 0.976700315 |
| wavelet.LHL.glrlm.RunLengthNonUniformity | 0.948591571 | 0.942692179 |
| wavelet.LHL.glrlm.RunLengthNonUniformityNormalized | 0.992900572 | 0.998058202 |
| wavelet.LHL.glrlm.RunPercentage | 0.997232221 | 0.952332818 |
| wavelet.LHL.glrlm.RunVariance | 0.997586484 | 0.952238868 |
| wavelet.LHL.glrlm.ShortRunEmphasis | 0.996516701 | 0.973455878 |
| wavelet.LHL.glrlm.ShortRunHighGrayLevelEmphasis | 0.997210435 | 0.965488851 |
| wavelet.LHL.glszm.GrayLevelNonUniformity | 0.804387462 | 0.820959966 |
| wavelet.LHL.glszm.GrayLevelNonUniformityNormalized | 0.994295193 | 0.998168428 |
| wavelet.LHL.glszm.GrayLevelVariance | 0.986099404 | 0.9933597 |
| wavelet.LHL.glszm.HighGrayLevelZoneEmphasis | 0.964766701 | 0.905871355 |
| wavelet.LHL.glszm.LargeAreaEmphasis | 0.844791498 | 0.870396996 |
| wavelet.LHL.glszm.LargeAreaHighGrayLevelEmphasis | 0.987568546 | 0.988938891 |
| wavelet.LHL.glszm.LargeAreaLowGrayLevelEmphasis | 0.847752053 | 0.955640463 |
| wavelet.LHL.glszm.LowGrayLevelZoneEmphasis | 0.868222703 | 0.993076299 |
| wavelet.LHL.glszm.SizeZoneNonUniformityNormalized | 0.994658994 | 0.99728211 |
| wavelet.LHL.glszm.SmallAreaEmphasis | 0.962139117 | 0.936854804 |
| wavelet.LHL.glszm.SmallAreaHighGrayLevelEmphasis | 0.958582225 | 0.9266901 |
| wavelet.LHL.glszm.ZoneEntropy | 0.926920798 | 0.919125388 |
| wavelet.LHL.glszm.ZonePercentage | 0.924732845 | 0.952799372 |
| wavelet.LHL.glszm.ZoneVariance | 0.984322374 | 0.877600845 |
| wavelet.LHL.ngtdm.Busyness | 0.98757903 | 0.988947212 |
| wavelet.LHL.ngtdm.Coarseness | 0.825524543 | 0.835496144 |
| wavelet.LHL.ngtdm.Complexity | 0.990947225 | 0.996623633 |
| wavelet.LHL.ngtdm.Contrast | 0.965439974 | 0.776245916 |
| wavelet.LHL.ngtdm.Strength | 0.863426069 | 0.937346676 |
| wavelet.LHH.firstorder.90Percentile | 0.988295917 | 0.972965075 |
| wavelet.LHH.firstorder.Energy | 0.985085395 | 0.970733191 |
| wavelet.LHH.firstorder.Entropy | 0.988793846 | 0.997870215 |
| wavelet.LHH.firstorder.InterquartileRange | 0.984746957 | 0.971970187 |
| wavelet.LHH.firstorder.Kurtosis | 0.988492867 | 0.968748473 |
| wavelet.LHH.firstorder.Maximum | 0.973831511 | 0.984570933 |
| wavelet.LHH.firstorder.Mean | 0.986581601 | 0.970355518 |
| wavelet.LHH.firstorder.Median | 0.768539844 | 0.83323931 |
| wavelet.LHH.firstorder.Minimum | 0.976067928 | 0.985339182 |
| wavelet.LHH.firstorder.Range | 0.939948062 | 0.785210235 |
| wavelet.LHH.firstorder.RobustMeanAbsoluteDeviation | 0.879598176 | 0.767373594 |
| wavelet.LHH.firstorder.RootMeanSquared | 0.98798617 | 0.969732677 |
| wavelet.LHH.firstorder.Skewness | 0.985052882 | 0.970576373 |
| wavelet.LHH.firstorder.TotalEnergy | 0.994147208 | 0.968127434 |
| wavelet.LHH.firstorder.Uniformity | 0.988793846 | 0.997870215 |
| wavelet.LHH.firstorder.Variance | 0.986150645 | 0.972471758 |
| wavelet.LHH.glcm.Autocorrelation | 0.986343118 | 0.966830822 |
| wavelet.LHH.glcm.ClusterProminence | 0.999999124 | 0.800513689 |
| wavelet.LHH.glcm.ClusterShade | 0.983915262 | 0.955945543 |
| wavelet.LHH.glcm.ClusterTendency | 0.992416931 | 0.976567492 |
| wavelet.LHH.glcm.Contrast | 0.988355227 | 0.961886684 |
| wavelet.LHH.glcm.Correlation | 0.987714822 | 0.959655588 |
| wavelet.LHH.glcm.DifferenceAverage | 0.899886632 | 0.884862553 |
| wavelet.LHH.glcm.DifferenceEntropy | 0.988776609 | 0.963190174 |
| wavelet.LHH.glcm.DifferenceVariance | 0.986646342 | 0.970719307 |
| wavelet.LHH.glcm.Id | 0.985409115 | 0.964240317 |
| wavelet.LHH.glcm.Idm | 0.98933786 | 0.966194384 |
| wavelet.LHH.glcm.Idmn | 0.989275395 | 0.965690492 |
| wavelet.LHH.glcm.Imc1 | 0.940056212 | 0.898528693 |
| wavelet.LHH.glcm.Imc2 | 0.994186061 | 0.994024094 |
| wavelet.LHH.glcm.InverseVariance | 0.987360862 | 0.995215303 |
| wavelet.LHH.glcm.JointAverage | 0.938506851 | 0.880915811 |
| wavelet.LHH.glcm.JointEnergy | 0.99999889 | 0.800583438 |
| wavelet.LHH.glcm.JointEntropy | 0.988380401 | 0.97516844 |
| wavelet.LHH.glcm.MaximumProbability | 0.984710719 | 0.995871308 |
| wavelet.LHH.glcm.MCC | 0.98782107 | 0.970196698 |
| wavelet.LHH.glcm.SumAverage | 0.991090441 | 0.982801169 |
| wavelet.LHH.glcm.SumEntropy | 0.999998939 | 0.800630155 |
| wavelet.LHH.glcm.SumSquares | 0.988009102 | 0.971265267 |
| wavelet.LHH.gldm.DependenceEntropy | 0.988151265 | 0.962418106 |
| wavelet.LHH.gldm.DependenceNonUniformity | 0.97756818 | 0.962526568 |
| wavelet.LHH.gldm.DependenceNonUniformityNormalized | 0.993356451 | 0.998305135 |
| wavelet.LHH.gldm.DependenceVariance | 0.982111007 | 0.971363599 |
| wavelet.LHH.gldm.GrayLevelNonUniformity | 0.96460438 | 0.982918377 |
| wavelet.LHH.gldm.GrayLevelVariance | 0.993405101 | 0.998386918 |
| wavelet.LHH.gldm.HighGrayLevelEmphasis | 0.985939952 | 0.965799248 |
| wavelet.LHH.gldm.LargeDependenceEmphasis | 0.999994747 | 0.803172275 |
| wavelet.LHH.gldm.LargeDependenceHighGrayLevelEmphasis | 0.984316351 | 0.96443677 |
| wavelet.LHH.gldm.LargeDependenceLowGrayLevelEmphasis | 0.998680006 | 0.783316894 |
| wavelet.LHH.gldm.LowGrayLevelEmphasis | 0.998832299 | 0.833143038 |
| wavelet.LHH.gldm.SmallDependenceEmphasis | 0.999955914 | 0.795678326 |
| wavelet.LHH.gldm.SmallDependenceHighGrayLevelEmphasis | 0.983873377 | 0.985022975 |
| wavelet.LHH.gldm.SmallDependenceLowGrayLevelEmphasis | 0.99756465 | 0.913825835 |
| wavelet.LHH.glrlm.GrayLevelNonUniformity | 0.988628762 | 0.758996034 |
| wavelet.LHH.glrlm.GrayLevelNonUniformityNormalized | 0.993551958 | 0.998518588 |
| wavelet.LHH.glrlm.GrayLevelVariance | 0.984012393 | 0.977116015 |
| wavelet.LHH.glrlm.HighGrayLevelRunEmphasis | 0.983900251 | 0.969883575 |
| wavelet.LHH.glrlm.LongRunEmphasis | 0.999993812 | 0.800310055 |
| wavelet.LHH.glrlm.LongRunHighGrayLevelEmphasis | 0.981464678 | 0.983071594 |
| wavelet.LHH.glrlm.LongRunLowGrayLevelEmphasis | 0.997870459 | 0.765671177 |
| wavelet.LHH.glrlm.LowGrayLevelRunEmphasis | 0.998203385 | 0.835285331 |
| wavelet.LHH.glrlm.RunEntropy | 0.999912136 | 0.791449196 |
| wavelet.LHH.glrlm.RunLengthNonUniformity | 0.883777605 | 0.972906474 |
| wavelet.LHH.glrlm.RunLengthNonUniformityNormalized | 0.992303575 | 0.998655906 |
| wavelet.LHH.glrlm.RunPercentage | 0.984540891 | 0.970772056 |
| wavelet.LHH.glrlm.RunVariance | 0.984291405 | 0.965453751 |
| wavelet.LHH.glrlm.ShortRunEmphasis | 0.979321017 | 0.982161109 |
| wavelet.LHH.glrlm.ShortRunHighGrayLevelEmphasis | 0.984193414 | 0.975241555 |
| wavelet.LHH.glrlm.ShortRunLowGrayLevelEmphasis | 0.999821823 | 0.822434886 |
| wavelet.LHH.glszm.GrayLevelNonUniformity | 0.999352586 | 0.778346361 |
| wavelet.LHH.glszm.GrayLevelNonUniformityNormalized | 0.993101378 | 0.997834453 |
| wavelet.LHH.glszm.GrayLevelVariance | 0.977789436 | 0.96311667 |
| wavelet.LHH.glszm.HighGrayLevelZoneEmphasis | 0.97830018 | 0.96479676 |
| wavelet.LHH.glszm.LargeAreaEmphasis | 0.999802007 | 0.768093767 |
| wavelet.LHH.glszm.LargeAreaHighGrayLevelEmphasis | 0.992668352 | 0.997877411 |
| wavelet.LHH.glszm.LargeAreaLowGrayLevelEmphasis | 0.995381055 | 0.988375183 |
| wavelet.LHH.glszm.LowGrayLevelZoneEmphasis | 0.988128093 | 0.968582883 |
| wavelet.LHH.glszm.SizeZoneNonUniformity | 0.99866174 | 0.763392922 |
| wavelet.LHH.glszm.SizeZoneNonUniformityNormalized | 0.993695711 | 0.998031117 |
| wavelet.LHH.glszm.SmallAreaEmphasis | 0.991401603 | 0.969333601 |
| wavelet.LHH.glszm.SmallAreaHighGrayLevelEmphasis | 0.991775516 | 0.970028619 |
| wavelet.LHH.glszm.SmallAreaLowGrayLevelEmphasis | 0.999584449 | 0.835437718 |
| wavelet.LHH.glszm.ZoneEntropy | 0.81998817 | 0.973849643 |
| wavelet.LHH.glszm.ZonePercentage | 0.935076572 | 0.91269348 |
| wavelet.LHH.glszm.ZoneVariance | 0.973348218 | 0.988838231 |
| wavelet.LHH.ngtdm.Busyness | 0.992671475 | 0.997878232 |
| wavelet.LHH.ngtdm.Coarseness | 0.990191555 | 0.959568259 |
| wavelet.LHH.ngtdm.Complexity | 0.989411653 | 0.997119735 |
| wavelet.LHH.ngtdm.Contrast | 0.89493563 | 0.826705109 |
| wavelet.LHH.ngtdm.Strength | 0.78878233 | 0.868261521 |
| wavelet.HLL.firstorder.10Percentile | 0.922457222 | 0.766245347 |
| wavelet.HLL.firstorder.90Percentile | 0.998912627 | 0.98712453 |
| wavelet.HLL.firstorder.Energy | 0.998092004 | 0.984415191 |
| wavelet.HLL.firstorder.Entropy | 0.997628485 | 0.995315014 |
| wavelet.HLL.firstorder.InterquartileRange | 0.999311208 | 0.983855556 |
| wavelet.HLL.firstorder.Kurtosis | 0.997308874 | 0.982521506 |
| wavelet.HLL.firstorder.Maximum | 0.953337325 | 0.991390426 |
| wavelet.HLL.firstorder.Mean | 0.998775024 | 0.985046497 |
| wavelet.HLL.firstorder.MeanAbsoluteDeviation | 0.995737538 | 0.954969158 |
| wavelet.HLL.firstorder.Median | 0.795811731 | 0.92401941 |
| wavelet.HLL.firstorder.Minimum | 0.774318091 | 0.769690265 |
| wavelet.HLL.firstorder.Range | 0.933651795 | 0.985022999 |
| wavelet.HLL.firstorder.RobustMeanAbsoluteDeviation | 0.983602623 | 0.985614995 |
| wavelet.HLL.firstorder.RootMeanSquared | 0.997787349 | 0.983744126 |
| wavelet.HLL.firstorder.Skewness | 0.999131236 | 0.98610821 |
| wavelet.HLL.firstorder.TotalEnergy | 0.876859591 | 0.907877236 |
| wavelet.HLL.firstorder.Uniformity | 0.997628485 | 0.995315014 |
| wavelet.HLL.firstorder.Variance | 0.999379548 | 0.980757308 |
| wavelet.HLL.glcm.Autocorrelation | 0.999135528 | 0.98054176 |
| wavelet.HLL.glcm.ClusterProminence | 0.974462542 | 0.999998932 |
| wavelet.HLL.glcm.ClusterShade | 0.996297362 | 0.984668145 |
| wavelet.HLL.glcm.ClusterTendency | 0.771169825 | 0.750162448 |
| wavelet.HLL.glcm.Contrast | 0.998667644 | 0.983102214 |
| wavelet.HLL.glcm.Correlation | 0.998977811 | 0.969184275 |
| wavelet.HLL.glcm.DifferenceEntropy | 0.998821604 | 0.96502077 |
| wavelet.HLL.glcm.DifferenceEntropy | 0.921759522 | 0.872366752 |
| wavelet.HLL.glcm.DifferenceVariance | 0.999248384 | 0.981184929 |
| wavelet.HLL.glcm.Id | 0.999147608 | 0.979052822 |
| wavelet.HLL.glcm.Idm | 0.99856515 | 0.961018866 |
| wavelet.HLL.glcm.Idmn | 0.998668974 | 0.962780723 |
| wavelet.HLL.glcm.Idn | 0.976646557 | 0.994179521 |
| wavelet.HLL.glcm.Imc1 | 0.975738304 | 0.991077484 |
| wavelet.HLL.glcm.Imc2 | 0.995828912 | 0.992694111 |
| wavelet.HLL.glcm.InverseVariance | 0.996924811 | 0.980316385 |
| wavelet.HLL.glcm.JointAverage | 0.988286135 | 0.877619144 |
| wavelet.HLL.glcm.JointEnergy | 0.970556458 | 0.999998538 |
| wavelet.HLL.glcm.JointEntropy | 0.99933184 | 0.982834691 |
| wavelet.HLL.glcm.MaximumProbability | 0.898214852 | 0.964505013 |
| wavelet.HLL.glcm.MCC | 0.999279369 | 0.983737167 |
| wavelet.HLL.glcm.SumAverage | 0.99909355 | 0.989115577 |
| wavelet.HLL.glcm.SumEntropy | 0.970562273 | 0.999998649 |
| wavelet.HLL.glcm.SumSquares | 0.999136138 | 0.989030471 |
| wavelet.HLL.gldm.DependenceEntropy | 0.998940708 | 0.97865125 |
| wavelet.HLL.gldm.DependenceNonUniformity | 0.997273361 | 0.997487272 |
| wavelet.HLL.gldm.DependenceNonUniformityNormalized | 0.990693903 | 0.998287101 |
| wavelet.HLL.gldm.DependenceVariance | 0.995223657 | 0.993022998 |
| wavelet.HLL.gldm.GrayLevelNonUniformity | 0.994829554 | 0.985810584 |
| wavelet.HLL.gldm.GrayLevelVariance | 0.990792972 | 0.998452685 |
| wavelet.HLL.gldm.HighGrayLevelEmphasis | 0.999150907 | 0.977145249 |
| wavelet.HLL.gldm.LargeDependenceEmphasis | 0.974767866 | 0.99999407 |
| wavelet.HLL.gldm.LargeDependenceHighGrayLevelEmphasis | 0.993970816 | 0.94131674 |
| wavelet.HLL.gldm.LargeDependenceLowGrayLevelEmphasis | 0.955547154 | 0.99600541 |
| wavelet.HLL.gldm.LowGrayLevelEmphasis | 0.980389414 | 0.999014775 |
| wavelet.HLL.gldm.SmallDependenceEmphasis | 0.975120056 | 0.999993905 |
| wavelet.HLL.gldm.SmallDependenceHighGrayLevelEmphasis | 0.995995191 | 0.968252116 |
| wavelet.HLL.gldm.SmallDependenceLowGrayLevelEmphasis | 0.992437753 | 0.995265449 |
| wavelet.HLL.glrlm.GrayLevelNonUniformity | 0.954345178 | 0.985755008 |
| wavelet.HLL.glrlm.GrayLevelNonUniformityNormalized | 0.992132355 | 0.998588673 |
| wavelet.HLL.glrlm.GrayLevelVariance | 0.999512602 | 0.988088512 |
| wavelet.HLL.glrlm.HighGrayLevelRunEmphasis | 0.999225098 | 0.982972655 |
| wavelet.HLL.glrlm.LongRunEmphasis | 0.974941565 | 0.999994632 |
| wavelet.HLL.glrlm.LongRunHighGrayLevelEmphasis | 0.993837521 | 0.958635214 |
| wavelet.HLL.glrlm.LongRunLowGrayLevelEmphasis | 0.950639667 | 0.995559091 |
| wavelet.HLL.glrlm.LowGrayLevelRunEmphasis | 0.981347493 | 0.99911765 |
| wavelet.HLL.glrlm.RunEntropy | 0.97476664 | 0.999987207 |
| wavelet.HLL.glrlm.RunLengthNonUniformity | 0.993094926 | 0.99781338 |
| wavelet.HLL.glrlm.RunLengthNonUniformityNormalized | 0.996710293 | 0.9959722 |
| wavelet.HLL.glrlm.RunPercentage | 0.995651917 | 0.956458649 |
| wavelet.HLL.glrlm.RunVariance | 0.994882013 | 0.950705409 |
| wavelet.HLL.glrlm.ShortRunEmphasis | 0.992125445 | 0.945789033 |
| wavelet.HLL.glrlm.ShortRunHighGrayLevelEmphasis | 0.995844202 | 0.966107248 |
| wavelet.HLL.glrlm.ShortRunLowGrayLevelEmphasis | 0.981917117 | 0.999501383 |
| wavelet.HLL.glszm.GrayLevelNonUniformity | 0.970275237 | 0.999556716 |
| wavelet.HLL.glszm.GrayLevelNonUniformityNormalized | 0.996395567 | 0.998217813 |
| wavelet.HLL.glszm.GrayLevelVariance | 0.990791472 | 0.973519117 |
| wavelet.HLL.glszm.HighGrayLevelZoneEmphasis | 0.997490722 | 0.982237998 |
| wavelet.HLL.glszm.LargeAreaEmphasis | 0.975151782 | 0.999944672 |
| wavelet.HLL.glszm.LargeAreaHighGrayLevelEmphasis | 0.983937709 | 0.996277545 |
| wavelet.HLL.glszm.LargeAreaLowGrayLevelEmphasis | 0.973261397 | 0.996741112 |
| wavelet.HLL.glszm.LowGrayLevelZoneEmphasis | 0.994439372 | 0.999121589 |
| wavelet.HLL.glszm.SizeZoneNonUniformity | 0.981103073 | 0.999733395 |
| wavelet.HLL.glszm.SizeZoneNonUniformityNormalized | 0.997804002 | 0.992421668 |
| wavelet.HLL.glszm.SmallAreaEmphasis | 0.992962297 | 0.994397508 |
| wavelet.HLL.glszm.SmallAreaHighGrayLevelEmphasis | 0.992279809 | 0.992453491 |
| wavelet.HLL.glszm.SmallAreaLowGrayLevelEmphasis | 0.96406761 | 0.999680018 |
| wavelet.HLL.glszm.ZoneEntropy | 0.985403317 | 0.998935768 |
| wavelet.HLL.glszm.ZonePercentage | 0.995250339 | 0.996563293 |
| wavelet.HLL.glszm.ZoneVariance | 0.996419545 | 0.979598026 |
| wavelet.HLL.ngtdm.Busyness | 0.983983591 | 0.996278909 |
| wavelet.HLL.ngtdm.Coarseness | 0.990012299 | 0.998945677 |
| wavelet.HLL.ngtdm.Complexity | 0.986954507 | 0.997773499 |
| wavelet.HLL.ngtdm.Contrast | 0.996994276 | 0.987411122 |
| wavelet.HLL.ngtdm.Strength | 0.971690741 | 0.993316564 |
| wavelet.HLH.firstorder.10Percentile | 0.980190538 | 0.978720269 |
| wavelet.HLH.firstorder.90Percentile | 0.995770345 | 0.951942341 |
| wavelet.HLH.firstorder.Energy | 0.995397513 | 0.949780037 |
| wavelet.HLH.firstorder.Entropy | 0.998197772 | 0.990725847 |
| wavelet.HLH.firstorder.InterquartileRange | 0.997566845 | 0.957952521 |
| wavelet.HLH.firstorder.Kurtosis | 0.993319736 | 0.952052537 |
| wavelet.HLH.firstorder.Maximum | 0.951597908 | 0.984027831 |
| wavelet.HLH.firstorder.Mean | 0.995567068 | 0.954151475 |
| wavelet.HLH.firstorder.MeanAbsoluteDeviation | 0.932957974 | 0.927608394 |
| wavelet.HLH.firstorder.Minimum | 0.982291011 | 0.997812416 |
| wavelet.HLH.firstorder.Range | 0.993405796 | 0.986827677 |
| wavelet.HLH.firstorder.RobustMeanAbsoluteDeviation | 0.977402513 | 0.968028766 |
| wavelet.HLH.firstorder.RootMeanSquared | 0.993878754 | 0.951256771 |
| wavelet.HLH.firstorder.Skewness | 0.996699685 | 0.957176035 |
| wavelet.HLH.firstorder.TotalEnergy | 0.968206779 | 0.979883864 |
| wavelet.HLH.firstorder.Uniformity | 0.998197772 | 0.990725847 |
| wavelet.HLH.firstorder.Variance | 0.997479276 | 0.956951462 |
| wavelet.HLH.glcm.Autocorrelation | 0.996956702 | 0.957934171 |
| wavelet.HLH.glcm.ClusterProminence | 0.99999989 | 0.944594674 |
| wavelet.HLH.glcm.ClusterShade | 0.997893568 | 0.977601212 |
| wavelet.HLH.glcm.ClusterTendency | 0.962655315 | 0.978897908 |
| wavelet.HLH.glcm.Contrast | 0.997878194 | 0.966551175 |
| wavelet.HLH.glcm.Correlation | 0.997780132 | 0.964224897 |
| wavelet.HLH.glcm.DifferenceAverage | 0.995114966 | 0.954798983 |
| wavelet.HLH.glcm.DifferenceEntropy | 0.99763664 | 0.958565289 |
| wavelet.HLH.glcm.DifferenceVariance | 0.997911599 | 0.9611779 |
| wavelet.HLH.glcm.Id | 0.997973171 | 0.967978469 |
| wavelet.HLH.glcm.Idm | 0.997495306 | 0.953910249 |
| wavelet.HLH.glcm.Idmn | 0.997532371 | 0.954941604 |
| wavelet.HLH.glcm.Idn | 0.933690869 | 0.785532502 |
| wavelet.HLH.glcm.Imc1 | 0.94199914 | 0.820332539 |
| wavelet.HLH.glcm.Imc2 | 0.9948035 | 0.913438443 |
| wavelet.HLH.glcm.InverseVariance | 0.996618892 | 0.933115861 |
| wavelet.HLH.glcm.JointAverage | 0.995908293 | 0.944548028 |
| wavelet.HLH.glcm.JointEnergy | 0.999999866 | 0.930489891 |
| wavelet.HLH.glcm.JointEntropy | 0.997818139 | 0.959185615 |
| wavelet.HLH.glcm.MaximumProbability | 0.92429288 | 0.932332665 |
| wavelet.HLH.glcm.MCC | 0.997865759 | 0.960237238 |
| wavelet.HLH.glcm.SumAverage | 0.996801435 | 0.970721531 |
| wavelet.HLH.glcm.SumEntropy | 0.99999986 | 0.930499477 |
| wavelet.HLH.glcm.SumSquares | 0.997942397 | 0.962331958 |
| wavelet.HLH.gldm.DependenceEntropy | 0.997839396 | 0.964711224 |
| wavelet.HLH.gldm.DependenceNonUniformity | 0.982116609 | 0.960862519 |
| wavelet.HLH.gldm.DependenceNonUniformityNormalized | 0.990324577 | 0.998619984 |
| wavelet.HLH.gldm.DependenceVariance | 0.982970427 | 0.963230338 |
| wavelet.HLH.gldm.GrayLevelNonUniformity | 0.988180023 | 0.949206208 |
| wavelet.HLH.gldm.GrayLevelVariance | 0.991865273 | 0.998840173 |
| wavelet.HLH.gldm.HighGrayLevelEmphasis | 0.997574637 | 0.964223514 |
| wavelet.HLH.gldm.LargeDependenceEmphasis | 0.99999969 | 0.944709334 |
| wavelet.HLH.gldm.LargeDependenceHighGrayLevelEmphasis | 0.986259714 | 0.943452033 |
| wavelet.HLH.gldm.LargeDependenceLowGrayLevelEmphasis | 0.999535241 | 0.939091788 |
| wavelet.HLH.gldm.LowGrayLevelEmphasis | 0.999831889 | 0.883983365 |
| wavelet.HLH.gldm.SmallDependenceEmphasis | 0.999996037 | 0.876482814 |
| wavelet.HLH.gldm.SmallDependenceHighGrayLevelEmphasis | 0.992394181 | 0.942645063 |
| wavelet.HLH.gldm.SmallDependenceLowGrayLevelEmphasis | 0.998141454 | 0.950837699 |
| wavelet.HLH.glrlm.GrayLevelNonUniformity | 0.990127474 | 0.817894234 |
| wavelet.HLH.glrlm.GrayLevelNonUniformityNormalized | 0.992203765 | 0.99883506 |
| wavelet.HLH.glrlm.GrayLevelVariance | 0.997747029 | 0.954396936 |
| wavelet.HLH.glrlm.HighGrayLevelRunEmphasis | 0.99782477 | 0.962357107 |
| wavelet.HLH.glrlm.LongRunEmphasis | 0.999999092 | 0.944876025 |
| wavelet.HLH.glrlm.LongRunHighGrayLevelEmphasis | 0.987712657 | 0.949453299 |
| wavelet.HLH.glrlm.LongRunLowGrayLevelEmphasis | 0.999416135 | 0.937995177 |
| wavelet.HLH.glrlm.LowGrayLevelRunEmphasis | 0.999726047 | 0.886401934 |
| wavelet.HLH.glrlm.RunEntropy | 0.999991574 | 0.873149642 |
| wavelet.HLH.glrlm.RunLengthNonUniformity | 0.998097948 | 0.970644717 |
| wavelet.HLH.glrlm.RunLengthNonUniformityNormalized | 0.994773379 | 0.997406278 |
| wavelet.HLH.glrlm.RunPercentage | 0.986659583 | 0.945320437 |
| wavelet.HLH.glrlm.RunVariance | 0.98646786 | 0.944889417 |
| wavelet.HLH.glrlm.ShortRunEmphasis | 0.986285858 | 0.955910637 |
| wavelet.HLH.glrlm.ShortRunHighGrayLevelEmphasis | 0.988173239 | 0.943943607 |
| wavelet.HLH.glrlm.ShortRunLowGrayLevelEmphasis | 0.999895119 | 0.947264744 |
| wavelet.HLH.glszm.GrayLevelNonUniformity | 0.999850665 | 0.866588566 |
| wavelet.HLH.glszm.GrayLevelNonUniformityNormalized | 0.99827359 | 0.981958439 |
| wavelet.HLH.glszm.GrayLevelVariance | 0.990470881 | 0.977871318 |
| wavelet.HLH.glszm.HighGrayLevelZoneEmphasis | 0.996746007 | 0.983747747 |
| wavelet.HLH.glszm.LargeAreaEmphasis | 0.999959696 | 0.947419483 |
| wavelet.HLH.glszm.LargeAreaHighGrayLevelEmphasis | 0.984960788 | 0.99254788 |
| wavelet.HLH.glszm.LargeAreaLowGrayLevelEmphasis | 0.971222429 | 0.995129103 |
| wavelet.HLH.glszm.LowGrayLevelZoneEmphasis | 0.990168632 | 0.983816521 |
| wavelet.HLH.glszm.SizeZoneNonUniformity | 0.999651323 | 0.852311365 |
| wavelet.HLH.glszm.SizeZoneNonUniformityNormalized | 0.998468017 | 0.988464051 |
| wavelet.HLH.glszm.SmallAreaEmphasis | 0.991009664 | 0.943317506 |
| wavelet.HLH.glszm.SmallAreaHighGrayLevelEmphasis | 0.991057045 | 0.941335673 |
| wavelet.HLH.glszm.SmallAreaLowGrayLevelEmphasis | 0.999573344 | 0.946038159 |
| wavelet.HLH.glszm.ZoneEntropy | 0.999815116 | 0.855709608 |
| wavelet.HLH.glszm.ZonePercentage | 0.994456034 | 0.954099494 |
| wavelet.HLH.glszm.ZoneVariance | 0.994163567 | 0.935473883 |
| wavelet.HLH.ngtdm.Busyness | 0.984969493 | 0.992562161 |
| wavelet.HLH.ngtdm.Coarseness | 0.987690214 | 0.965696443 |
| wavelet.HLH.ngtdm.Complexity | 0.987312887 | 0.998556453 |
| wavelet.HLH.ngtdm.Contrast | 0.988876878 | 0.936758344 |
| wavelet.HLH.ngtdm.Strength | 0.923782567 | 0.764355353 |
| wavelet.HHL.firstorder.10Percentile | 0.963274374 | 0.888797805 |
| wavelet.HHL.firstorder.90Percentile | 0.99883542 | 0.987297008 |
| wavelet.HHL.firstorder.Energy | 0.999043972 | 0.987489798 |
| wavelet.HHL.firstorder.Entropy | 0.996143088 | 0.996669948 |
| wavelet.HHL.firstorder.InterquartileRange | 0.999317725 | 0.990846962 |
| wavelet.HHL.firstorder.Kurtosis | 0.99732792 | 0.983587634 |
| wavelet.HHL.firstorder.Maximum | 0.942992573 | 0.974251701 |
| wavelet.HHL.firstorder.Mean | 0.998858073 | 0.988254996 |
| wavelet.HHL.firstorder.MeanAbsoluteDeviation | 0.964222679 | 0.930042398 |
| wavelet.HHL.firstorder.Median | 0.930957718 | 0.840127323 |
| wavelet.HHL.firstorder.Minimum | 0.848063332 | 0.775148052 |
| wavelet.HHL.firstorder.Range | 0.904859746 | 0.927409818 |
| wavelet.HHL.firstorder.RobustMeanAbsoluteDeviation | 0.961106088 | 0.954103036 |
| wavelet.HHL.firstorder.RootMeanSquared | 0.997827624 | 0.984748494 |
| wavelet.HHL.firstorder.Skewness | 0.999189247 | 0.991123866 |
| wavelet.HHL.firstorder.TotalEnergy | 0.84117963 | 0.981964934 |
| wavelet.HHL.firstorder.Uniformity | 0.996143088 | 0.996669948 |
| wavelet.HHL.firstorder.Variance | 0.999513336 | 0.988241591 |
| wavelet.HHL.glcm.Autocorrelation | 0.999301572 | 0.986190575 |
| wavelet.HHL.glcm.ClusterProminence | 0.870197988 | 0.87270984 |
| wavelet.HHL.glcm.ClusterShade | 0.997940144 | 0.982983129 |
| wavelet.HHL.glcm.ClusterTendency | 0.908192941 | 0.960893727 |
| wavelet.HHL.glcm.Contrast | 0.999250555 | 0.981919764 |
| wavelet.HHL.glcm.Correlation | 0.999375221 | 0.984685442 |
| wavelet.HHL.glcm.DifferenceEntropy | 0.999571866 | 0.985681456 |
| wavelet.HHL.glcm.DifferenceVariance | 0.999464487 | 0.99237431 |
| wavelet.HHL.glcm.Id | 0.999080668 | 0.989654527 |
| wavelet.HHL.glcm.Idm | 0.99966929 | 0.986680818 |
| wavelet.HHL.glcm.Idmn | 0.999665021 | 0.98650858 |
| wavelet.HHL.glcm.Imc1 | 0.949976819 | 0.862714525 |
| wavelet.HHL.glcm.Imc2 | 0.993416923 | 0.97860167 |
| wavelet.HHL.glcm.InverseVariance | 0.995662282 | 0.96502855 |
| wavelet.HHL.glcm.JointAverage | 0.997800356 | 0.966554957 |
| wavelet.HHL.glcm.JointEnergy | 0.833319073 | 0.834749224 |
| wavelet.HHL.glcm.JointEntropy | 0.999656373 | 0.989672885 |
| wavelet.HHL.glcm.MaximumProbability | 0.7780659 | 0.808843282 |
| wavelet.HHL.glcm.MCC | 0.999483224 | 0.990650291 |
| wavelet.HHL.glcm.SumAverage | 0.999387035 | 0.993502825 |
| wavelet.HHL.glcm.SumEntropy | 0.833321746 | 0.834713325 |
| wavelet.HHL.glcm.SumSquares | 0.999411219 | 0.990032017 |
| wavelet.HHL.gldm.DependenceEntropy | 0.999270053 | 0.986172286 |
| wavelet.HHL.gldm.DependenceNonUniformity | 0.998969795 | 0.994360155 |
| wavelet.HHL.gldm.DependenceNonUniformityNormalized | 0.989307293 | 0.998139503 |
| wavelet.HHL.gldm.DependenceVariance | 0.999008699 | 0.99580687 |
| wavelet.HHL.gldm.GrayLevelNonUniformity | 0.998697565 | 0.997643185 |
| wavelet.HHL.gldm.GrayLevelVariance | 0.99119927 | 0.998412334 |
| wavelet.HHL.gldm.HighGrayLevelEmphasis | 0.999204955 | 0.98468176 |
| wavelet.HHL.gldm.LargeDependenceEmphasis | 0.873379615 | 0.876025926 |
| wavelet.HHL.gldm.LargeDependenceHighGrayLevelEmphasis | 0.998473649 | 0.982909294 |
| wavelet.HHL.gldm.LargeDependenceLowGrayLevelEmphasis | 0.93762312 | 0.816128809 |
| wavelet.HHL.gldm.SmallDependenceHighGrayLevelEmphasis | 0.997196488 | 0.992490363 |
| wavelet.HHL.gldm.SmallDependenceLowGrayLevelEmphasis | 0.979184 | 0.96884343 |
| wavelet.HHL.glrlm.GrayLevelNonUniformityNormalized | 0.991871345 | 0.99849044 |
| wavelet.HHL.glrlm.GrayLevelVariance | 0.99943909 | 0.992434578 |
| wavelet.HHL.glrlm.GrayLevelVariance | 0.841736587 | 0.975873789 |
| wavelet.HHL.glrlm.HighGrayLevelRunEmphasis | 0.999060413 | 0.988678963 |
| wavelet.HHL.glrlm.LongRunEmphasis | 0.874572193 | 0.877206333 |
| wavelet.HHL.glrlm.LongRunHighGrayLevelEmphasis | 0.997931023 | 0.98595914 |
| wavelet.HHL.glrlm.LowGrayLevelRunEmphasis | 0.999920064 | 0.846133516 |
| wavelet.HHL.glrlm.RunEntropy | 0.936730601 | 0.908298212 |
| wavelet.HHL.glrlm.RunLengthNonUniformity | 0.994123178 | 0.997618322 |
| wavelet.HHL.glrlm.RunLengthNonUniformityNormalized | 0.994884388 | 0.998098486 |
| wavelet.HHL.glrlm.RunPercentage | 0.998838923 | 0.984160225 |
| wavelet.HHL.glrlm.RunVariance | 0.998670095 | 0.984467375 |
| wavelet.HHL.glrlm.ShortRunEmphasis | 0.99777177 | 0.983255363 |
| wavelet.HHL.glrlm.ShortRunHighGrayLevelEmphasis | 0.998628032 | 0.986733673 |
| wavelet.HHL.glrlm.ShortRunLowGrayLevelEmphasis | 0.913035866 | 0.909828938 |
| wavelet.HHL.glszm.GrayLevelNonUniformity | 0.97690103 | 0.780674053 |
| wavelet.HHL.glszm.GrayLevelNonUniformityNormalized | 0.996392522 | 0.999157347 |
| wavelet.HHL.glszm.GrayLevelVariance | 0.993769828 | 0.992390577 |
| wavelet.HHL.glszm.HighGrayLevelZoneEmphasis | 0.992327979 | 0.992433748 |
| wavelet.HHL.glszm.LargeAreaEmphasis | 0.885161168 | 0.885472825 |
| wavelet.HHL.glszm.LargeAreaHighGrayLevelEmphasis | 0.985286298 | 0.997699462 |
| wavelet.HHL.glszm.LargeAreaLowGrayLevelEmphasis | 0.913670572 | 0.918231985 |
| wavelet.HHL.glszm.LowGrayLevelZoneEmphasis | 0.807247924 | 0.797934569 |
| wavelet.HHL.glszm.SizeZoneNonUniformity | 0.750075825 | 0.794831687 |
| wavelet.HHL.glszm.SizeZoneNonUniformityNormalized | 0.997398883 | 0.997734751 |
| wavelet.HHL.glszm.SmallAreaEmphasis | 0.971895568 | 0.992758221 |
| wavelet.HHL.glszm.SmallAreaHighGrayLevelEmphasis | 0.972423698 | 0.987859061 |
| wavelet.HHL.glszm.SmallAreaLowGrayLevelEmphasis | 0.85710024 | 0.850051626 |
| wavelet.HHL.glszm.ZoneEntropy | 0.815508728 | 0.76804047 |
| wavelet.HHL.glszm.ZonePercentage | 0.991763809 | 0.996888322 |
| wavelet.HHL.glszm.ZoneVariance | 0.996329043 | 0.998391607 |
| wavelet.HHL.ngtdm.Busyness | 0.985286723 | 0.997699087 |
| wavelet.HHL.ngtdm.Coarseness | 0.816385157 | 0.824810792 |
| wavelet.HHL.ngtdm.Complexity | 0.98746902 | 0.998523943 |
| wavelet.HHL.ngtdm.Contrast | 0.921852421 | 0.956253275 |
| wavelet.HHL.ngtdm.Strength | 0.905947873 | 0.836211564 |
| wavelet.HHH.firstorder.10Percentile | 0.764742047 | 0.894937214 |
| wavelet.HHH.firstorder.90Percentile | 0.994714231 | 0.945556987 |
| wavelet.HHH.firstorder.Energy | 0.995434149 | 0.947988404 |
| wavelet.HHH.firstorder.Entropy | 0.995830828 | 0.990939451 |
| wavelet.HHH.firstorder.InterquartileRange | 0.998438289 | 0.945743529 |
| wavelet.HHH.firstorder.Kurtosis | 0.991446849 | 0.952656405 |
| wavelet.HHH.firstorder.Maximum | 0.975433775 | 0.98796674 |
| wavelet.HHH.firstorder.Mean | 0.994866185 | 0.948790324 |
| wavelet.HHH.firstorder.MeanAbsoluteDeviation | 0.999731089 | 0.968105776 |
| wavelet.HHH.firstorder.Median | 0.782964614 | 0.967456739 |
| wavelet.HHH.firstorder.Minimum | 0.977495789 | 0.948681606 |
| wavelet.HHH.firstorder.Range | 0.983620628 | 0.962683769 |
| wavelet.HHH.firstorder.RobustMeanAbsoluteDeviation | 0.994967308 | 0.965869848 |
| wavelet.HHH.firstorder.RootMeanSquared | 0.992517761 | 0.950272629 |
| wavelet.HHH.firstorder.Skewness | 0.996598278 | 0.94797909 |
| wavelet.HHH.firstorder.TotalEnergy | 0.98450269 | 0.950961405 |
| wavelet.HHH.firstorder.Uniformity | 0.995830828 | 0.990939451 |
| wavelet.HHH.firstorder.Variance | 0.998639753 | 0.943844753 |
| wavelet.HHH.glcm.Autocorrelation | 0.997390346 | 0.944739265 |
| wavelet.HHH.glcm.ClusterProminence | 0.99999992 | 0.999999764 |
| wavelet.HHH.glcm.ClusterShade | 0.999032756 | 0.945673272 |
| wavelet.HHH.glcm.ClusterTendency | 0.992792525 | 0.990569411 |
| wavelet.HHH.glcm.Contrast | 0.999014807 | 0.946047244 |
| wavelet.HHH.glcm.Correlation | 0.999020921 | 0.946206416 |
| wavelet.HHH.glcm.DifferenceAverage | 0.991129676 | 0.965637546 |
| wavelet.HHH.glcm.DifferenceEntropy | 0.998991361 | 0.947224112 |
| wavelet.HHH.glcm.DifferenceVariance | 0.998866255 | 0.952172293 |
| wavelet.HHH.glcm.Id | 0.999030372 | 0.948585539 |
| wavelet.HHH.glcm.Idm | 0.998956053 | 0.948308394 |
| wavelet.HHH.glcm.Idmn | 0.998963442 | 0.947912102 |
| wavelet.HHH.glcm.Idn | 0.999719972 | 0.986361256 |
| wavelet.HHH.glcm.Imc1 | 0.999761055 | 0.989340186 |
| wavelet.HHH.glcm.Imc2 | 0.982837044 | 0.779951994 |
| wavelet.HHH.glcm.InverseVariance | 0.993618833 | 0.840108 |
| wavelet.HHH.glcm.JointAverage | 0.979703873 | 0.996134201 |
| wavelet.HHH.glcm.JointEnergy | 0.99999993 | 0.999999751 |
| wavelet.HHH.glcm.JointEntropy | 0.999055639 | 0.951683131 |
| wavelet.HHH.glcm.MaximumProbability | 0.999832308 | 0.980520847 |
| wavelet.HHH.glcm.MCC | 0.998849334 | 0.950821048 |
| wavelet.HHH.glcm.SumAverage | 0.998630042 | 0.964628441 |
| wavelet.HHH.glcm.SumEntropy | 0.999999924 | 0.999999715 |
| wavelet.HHH.glcm.SumSquares | 0.998888609 | 0.951666649 |
| wavelet.HHH.gldm.DependenceEntropy | 0.999056453 | 0.946504682 |
| wavelet.HHH.gldm.DependenceNonUniformity | 0.983383991 | 0.930858812 |
| wavelet.HHH.gldm.DependenceNonUniformityNormalized | 0.991290389 | 0.998509958 |
| wavelet.HHH.gldm.DependenceVariance | 0.99000126 | 0.946534347 |
| wavelet.HHH.gldm.GrayLevelNonUniformity | 0.989462209 | 0.933650859 |
| wavelet.HHH.gldm.GrayLevelVariance | 0.992324511 | 0.998829355 |
| wavelet.HHH.gldm.HighGrayLevelEmphasis | 0.998738647 | 0.94361917 |
| wavelet.HHH.gldm.LargeDependenceEmphasis | 0.999999822 | 0.999993559 |
| wavelet.HHH.gldm.LargeDependenceHighGrayLevelEmphasis | 0.99104695 | 0.942042569 |
| wavelet.HHH.gldm.LargeDependenceLowGrayLevelEmphasis | 0.999625788 | 0.998183222 |
| wavelet.HHH.gldm.LowGrayLevelEmphasis | 0.999875763 | 0.99910521 |
| wavelet.HHH.gldm.SmallDependenceEmphasis | 0.999994413 | 0.999973679 |
| wavelet.HHH.gldm.SmallDependenceHighGrayLevelEmphasis | 0.992786266 | 0.944584167 |
| wavelet.HHH.gldm.SmallDependenceLowGrayLevelEmphasis | 0.996050076 | 0.974765643 |
| wavelet.HHH.glrlm.GrayLevelNonUniformity | 0.996031966 | 0.979134922 |
| wavelet.HHH.glrlm.GrayLevelNonUniformityNormalized | 0.992677656 | 0.99883975 |
| wavelet.HHH.glrlm.GrayLevelVariance | 0.998520744 | 0.945621932 |
| wavelet.HHH.glrlm.HighGrayLevelRunEmphasis | 0.998702517 | 0.944242634 |
| wavelet.HHH.glrlm.LongRunEmphasis | 0.99999968 | 0.999992074 |
| wavelet.HHH.glrlm.LongRunHighGrayLevelEmphasis | 0.992607973 | 0.956391283 |
| wavelet.HHH.glrlm.LongRunLowGrayLevelEmphasis | 0.999562279 | 0.997842207 |
| wavelet.HHH.glrlm.LowGrayLevelRunEmphasis | 0.999853404 | 0.99918928 |
| wavelet.HHH.glrlm.RunEntropy | 0.999989184 | 0.999935023 |
| wavelet.HHH.glrlm.RunLengthNonUniformity | 0.993183482 | 0.9546597 |
| wavelet.HHH.glrlm.RunLengthNonUniformityNormalized | 0.994078759 | 0.997485671 |
| wavelet.HHH.glrlm.RunPercentage | 0.990906085 | 0.937590935 |
| wavelet.HHH.glrlm.RunVariance | 0.991097064 | 0.940349812 |
| wavelet.HHH.glrlm.ShortRunEmphasis | 0.992754293 | 0.963121007 |
| wavelet.HHH.glrlm.ShortRunHighGrayLevelEmphasis | 0.991596582 | 0.940303907 |
| wavelet.HHH.glrlm.ShortRunLowGrayLevelEmphasis | 0.999899588 | 0.999298135 |
| wavelet.HHH.glszm.GrayLevelNonUniformity | 0.999909124 | 0.999203307 |
| wavelet.HHH.glszm.GrayLevelNonUniformityNormalized | 0.996245775 | 0.992058989 |
| wavelet.HHH.glszm.GrayLevelVariance | 0.990488084 | 0.959382555 |
| wavelet.HHH.glszm.HighGrayLevelZoneEmphasis | 0.991025896 | 0.967127835 |
| wavelet.HHH.glszm.LargeAreaEmphasis | 0.99982999 | 0.999864109 |
| wavelet.HHH.glszm.LargeAreaHighGrayLevelEmphasis | 0.987700838 | 0.995408202 |
| wavelet.HHH.glszm.LargeAreaLowGrayLevelEmphasis | 0.988351073 | 0.996242077 |
| wavelet.HHH.glszm.LowGrayLevelZoneEmphasis | 0.991108521 | 0.996804454 |
| wavelet.HHH.glszm.SizeZoneNonUniformity | 0.999876311 | 0.999776564 |
| wavelet.HHH.glszm.SizeZoneNonUniformityNormalized | 0.995918459 | 0.99263881 |
| wavelet.HHH.glszm.SmallAreaEmphasis | 0.974035506 | 0.929817818 |
| wavelet.HHH.glszm.SmallAreaHighGrayLevelEmphasis | 0.972326871 | 0.923785034 |
| wavelet.HHH.glszm.SmallAreaLowGrayLevelEmphasis | 0.998361475 | 0.999143704 |
| wavelet.HHH.glszm.ZoneEntropy | 0.99952769 | 0.999598773 |
| wavelet.HHH.glszm.ZonePercentage | 0.993428008 | 0.934201414 |
| wavelet.HHH.glszm.ZoneVariance | 0.994405653 | 0.944631729 |
| wavelet.HHH.ngtdm.Busyness | 0.987694074 | 0.995416949 |
| wavelet.HHH.ngtdm.Coarseness | 0.992982809 | 0.995765618 |
| wavelet.HHH.ngtdm.Complexity | 0.987831422 | 0.998546653 |
| wavelet.HHH.ngtdm.Contrast | 0.999989761 | 0.966873544 |
| wavelet.HHH.ngtdm.Strength | 0.999324355 | 0.991128399 |
| wavelet.LLL.firstorder.10Percentile | 0.995059065 | 0.988116592 |
| wavelet.LLL.firstorder.90Percentile | 0.998503905 | 0.996107588 |
| wavelet.LLL.firstorder.Energy | 0.984474116 | 0.981595569 |
| wavelet.LLL.firstorder.Entropy | 0.987876323 | 0.997171537 |
| wavelet.LLL.firstorder.InterquartileRange | 0.920531428 | 0.950747246 |
| wavelet.LLL.firstorder.Kurtosis | 0.960368385 | 0.967308869 |
| wavelet.LLL.firstorder.Maximum | 0.802211707 | 0.98235909 |
| wavelet.LLL.firstorder.Mean | 0.899361468 | 0.940180194 |
| wavelet.LLL.firstorder.Median | 0.995970713 | 0.991488882 |
| wavelet.LLL.firstorder.Minimum | 0.997165819 | 0.993624329 |
| wavelet.LLL.firstorder.Range | 0.997162936 | 0.978246022 |
| wavelet.LLL.firstorder.RobustMeanAbsoluteDeviation | 0.986329524 | 0.865271399 |
| wavelet.LLL.firstorder.RootMeanSquared | 0.950588865 | 0.961919305 |
| wavelet.LLL.firstorder.Skewness | 0.99346392 | 0.989325805 |
| wavelet.LLL.firstorder.TotalEnergy | 0.94293873 | 0.993873436 |
| wavelet.LLL.firstorder.Uniformity | 0.987876325 | 0.997171537 |
| wavelet.LLL.firstorder.Variance | 0.955501835 | 0.961333277 |
| wavelet.LLL.glcm.Autocorrelation | 0.848795355 | 0.929498198 |
| wavelet.LLL.glcm.ClusterProminence | 0.993902973 | 0.927824759 |
| wavelet.LLL.glcm.ClusterShade | 0.772377116 | 0.929090952 |
| wavelet.LLL.glcm.ClusterTendency | 0.893605411 | 0.980946015 |
| wavelet.LLL.glcm.Contrast | 0.894276224 | 0.945190556 |
| wavelet.LLL.glcm.Correlation | 0.934850358 | 0.877566071 |
| wavelet.LLL.glcm.DifferenceAverage | 0.978625363 | 0.989103695 |
| wavelet.LLL.glcm.DifferenceEntropy | 0.970961654 | 0.936885499 |
| wavelet.LLL.glcm.DifferenceVariance | 0.960653229 | 0.938997467 |
| wavelet.LLL.glcm.Id | 0.878539594 | 0.839111348 |
| wavelet.LLL.glcm.Idm | 0.986304343 | 0.972168811 |
| wavelet.LLL.glcm.Idmn | 0.986090585 | 0.970849675 |
| wavelet.LLL.glcm.Idn | 0.914343583 | 0.91028498 |
| wavelet.LLL.glcm.Imc1 | 0.93040103 | 0.948863866 |
| wavelet.LLL.glcm.Imc2 | 0.981172536 | 0.989671934 |
| wavelet.LLL.glcm.InverseVariance | 0.982084214 | 0.98944309 |
| wavelet.LLL.glcm.JointEnergy | 0.995509686 | 0.942049174 |
| wavelet.LLL.glcm.JointEntropy | 0.984349987 | 0.980478401 |
| wavelet.LLL.glcm.MaximumProbability | 0.966021449 | 0.940649563 |
| wavelet.LLL.glcm.MCC | 0.964857415 | 0.952878973 |
| wavelet.LLL.glcm.SumAverage | 0.957760676 | 0.981035114 |
| wavelet.LLL.glcm.SumEntropy | 0.995552381 | 0.94452992 |
| wavelet.LLL.glcm.SumSquares | 0.945517935 | 0.958546367 |
| wavelet.LLL.gldm.DependenceEntropy | 0.885344611 | 0.948089436 |
| wavelet.LLL.gldm.DependenceNonUniformity | 0.929820425 | 0.980960131 |
| wavelet.LLL.gldm.DependenceNonUniformityNormalized | 0.993300753 | 0.99879384 |
| wavelet.LLL.gldm.DependenceVariance | 0.994666475 | 0.985096899 |
| wavelet.LLL.gldm.GrayLevelNonUniformity | 0.991001495 | 0.995888745 |
| wavelet.LLL.gldm.GrayLevelVariance | 0.99361248 | 0.995360034 |
| wavelet.LLL.gldm.HighGrayLevelEmphasis | 0.849331863 | 0.930016008 |
| wavelet.LLL.gldm.LargeDependenceEmphasis | 0.991081395 | 0.926336049 |
| wavelet.LLL.gldm.LargeDependenceHighGrayLevelEmphasis | 0.990522445 | 0.990585403 |
| wavelet.LLL.gldm.LargeDependenceLowGrayLevelEmphasis | 0.998363612 | 0.955954514 |
| wavelet.LLL.gldm.LowGrayLevelEmphasis | 0.984890727 | 0.998200593 |
| wavelet.LLL.gldm.SmallDependenceEmphasis | 0.987830352 | 0.987673134 |
| wavelet.LLL.gldm.SmallDependenceHighGrayLevelEmphasis | 0.973977617 | 0.940603793 |
| wavelet.LLL.gldm.SmallDependenceLowGrayLevelEmphasis | 0.942786123 | 0.874571877 |
| wavelet.LLL.glrlm.GrayLevelNonUniformity | 0.986739034 | 0.915479055 |
| wavelet.LLL.glrlm.GrayLevelNonUniformityNormalized | 0.99377169 | 0.996163639 |
| wavelet.LLL.glrlm.GrayLevelVariance | 0.924026512 | 0.94782178 |
| wavelet.LLL.glrlm.HighGrayLevelRunEmphasis | 0.78352774 | 0.916584494 |
| wavelet.LLL.glrlm.LongRunEmphasis | 0.9869222 | 0.9181135 |
| wavelet.LLL.glrlm.LongRunHighGrayLevelEmphasis | 0.989437164 | 0.994457201 |
| wavelet.LLL.glrlm.LongRunLowGrayLevelEmphasis | 0.994749411 | 0.930614499 |
| wavelet.LLL.glrlm.LowGrayLevelRunEmphasis | 0.982906442 | 0.997391915 |
| wavelet.LLL.glrlm.RunEntropy | 0.988953238 | 0.979371143 |
| wavelet.LLL.glrlm.RunLengthNonUniformity | 0.873090067 | 0.9760998 |
| wavelet.LLL.glrlm.RunLengthNonUniformityNormalized | 0.99045867 | 0.998463319 |
| wavelet.LLL.glrlm.RunPercentage | 0.987450865 | 0.975249669 |
| wavelet.LLL.glrlm.RunVariance | 0.990226855 | 0.983207879 |
| wavelet.LLL.glrlm.ShortRunEmphasis | 0.987542965 | 0.995908232 |
| wavelet.LLL.glrlm.ShortRunHighGrayLevelEmphasis | 0.988235911 | 0.980789338 |
| wavelet.LLL.glrlm.ShortRunLowGrayLevelEmphasis | 0.983024303 | 0.911844212 |
| wavelet.LLL.glszm.GrayLevelNonUniformity | 0.990549873 | 0.963388921 |
| wavelet.LLL.glszm.GrayLevelNonUniformityNormalized | 0.985842835 | 0.992006244 |
| wavelet.LLL.glszm.GrayLevelVariance | 0.900579268 | 0.993735666 |
| wavelet.LLL.glszm.HighGrayLevelZoneEmphasis | 0.986584973 | 0.965349709 |
| wavelet.LLL.glszm.LargeAreaEmphasis | 0.796677225 | 0.862484053 |
| wavelet.LLL.glszm.LargeAreaHighGrayLevelEmphasis | 0.984879264 | 0.982701763 |
| wavelet.LLL.glszm.LargeAreaLowGrayLevelEmphasis | 0.993998918 | 0.994332141 |
| wavelet.LLL.glszm.LowGrayLevelZoneEmphasis | 0.942288184 | 0.987356833 |
| wavelet.LLL.glszm.SizeZoneNonUniformity | 0.930058167 | 0.942958476 |
| wavelet.LLL.glszm.SizeZoneNonUniformityNormalized | 0.984413951 | 0.99122 |
| wavelet.LLL.glszm.SmallAreaEmphasis | 0.755803489 | 0.913108143 |
| wavelet.LLL.glszm.SmallAreaHighGrayLevelEmphasis | 0.79576783 | 0.927165031 |
| wavelet.LLL.glszm.SmallAreaLowGrayLevelEmphasis | 0.874800699 | 0.907669314 |
| wavelet.LLL.glszm.ZoneEntropy | 0.916707081 | 0.959936068 |
| wavelet.LLL.glszm.ZonePercentage | 0.939572319 | 0.780457245 |
| wavelet.LLL.glszm.ZoneVariance | 0.946376824 | 0.942110366 |
| wavelet.LLL.ngtdm.Busyness | 0.985004677 | 0.982810578 |
| wavelet.LLL.ngtdm.Coarseness | 0.967978797 | 0.978837141 |
| wavelet.LLL.ngtdm.Complexity | 0.993742683 | 0.995719691 |
| wavelet.LLL.ngtdm.Contrast | 0.81089618 | 0.949443478 |
| wavelet.LLL.ngtdm.Strength | 0.899328533 | 0.937069218 |
